# Inhibition of CHI3L1 decreases N-cadherin and VCAM-1 levels in glioblastoma

**DOI:** 10.1007/s43440-024-00677-3

**Published:** 2024-11-14

**Authors:** Agnieszka Rusak, Marlena Gąsior-Głogowska, Azzurra Sargenti, Edward Krzyżak, Krzysztof Kotowski, Monika Mrozowska, Tomasz Górnicki, Krzysztof Kujawa, Piotr Dzięgiel

**Affiliations:** 1 Division of Histology and Embryology, Department of Human Morphology and Embryology, Wroclaw Medical University, T. Chałubińskiego 6a, Wroclaw, 50-368 Poland; 2 Department of Biomedical Engineering, Faculty of Fundamental Problems of Technology, Wroclaw University of Science and Technology, 27, S. Wyspiańskiego, Wroclaw, 50-370 Poland; 3 CellDynamics Srl, Via Paolo Nanni Costa 20, Bologna, 40133 Italy; 4 Department of Basic Chemical Sciences, Faculty of Pharmacy, Wroclaw Medical University, Borowska 211A, Wroclaw, 50-556 Poland; 5 Statistical Analysis Centre, Wroclaw Medical University, K. Marcinkowskiego 2-6, Wroclaw, 50-368 Poland; 6 Department of Physiotherapy, University School of Physical Education, I. Paderewskiego 35, Wroclaw, 51-612 Poland

**Keywords:** CHI3L1, Glioblastoma, N-cadherin, VCAM-1, Physical cytometry

## Abstract

**Background:**

The protein CHI3L1 contributes to cancer development by several mechanisms, including stimulation of angiogenesis and invasion as well as immunomodulatory effects. These properties make it a potential target for the development of targeted therapies in precision medicine. In this context, the particular potential of CHI3L1 inhibition could be considered in glioblastoma multiforme (GBM), whose tumors exhibit high levels of angiogenesis and increased CHI3L1 expression. This study aims to investigate whether inhibition of CHI3L1 in spheroids used as a GBM model affects the mechanisms of invasiveness;

**Methods:**

We analyzed the interactions between CHI3L1 and the inhibitor G721-0282 in molecular docking and molecular dynamics (in silico) and infrared spectroscopy. Uptake of G721-0282 in GBM spheroids was measured using a label-free physical cytometer. Changes in E-, N- and VE-cadherins, VCAM-1, and EGFR were analyzed by immunohistochemical reactions, Western blot, and ddPCR methods in U-87 MG cells and GBM spheroids consisting of U-87 MG glioblastoma cells, HMEC-1 endothelial cells and macrophages;

**Results:**

A direct interaction between CHI3L1 and G721-0282 was confirmed. G721-0282 decreased N-cadherins and VCAM-1 in GBM spheroids, but the changes in the 2D model of U-87 MG glioblastoma cells were different;

**Conclusion:**

Inhibition of CHI3L1 has the potential to reduce the invasiveness of GBM tumors. The 3D model of GBM spheroids is of great significance for investigating changes in membrane proteins and the tumor microenvironment.

**Supplementary Information:**

The online version contains supplementary material available at 10.1007/s43440-024-00677-3.

## Introduction

The treatment of glioblastoma multiforme (GBM) remains a clinical challenge due to several key features of this cancer. These include its high degree of differentiation, its ability to develop resistance to drugs and radiotherapy, the blood-brain barrier (BBB) that restricts drug administration, limited resection options in the brain, and rapid recurrence [1–3]. In addition, the generally poor condition of patients often makes surgical intervention unfeasible. Currently, the treatment regimen for most GBM patients includes surgical resection, chemotherapy with temozolomide (TMZ), and radiotherapy [4–7]. Nevertheless, the statistics are far from satisfactoryconsidering that the overall survival of patients is only 8 months, and only 8% of patients survive 5 years after diagnosis [8].

The inhibition of angiogenesis, the process of new blood vessel development, is a potential target of great clinical importance. However, the expected results were not achieved. Bevacizumab, an anti-VEGF-A monoclonal antibody, is currently used in combination therapy, mainly with temozolomide, for the treatment of GBM [4, 6]. One of the proteins involved in angiogenesis in most cancers is CHI3L1 (YKL-40), which remains a potential candidate for anti-angiogenesis treatment. CHI3L1 belongs to the family of chitinase-like proteins and has no enzymatic activity. Elevated levels of CHI3L1 are a common feature in various cancers, including breast, lung, ovarian cancer, and glioblastoma, as well as in inflammatory diseases such as multiple sclerosis (MS), Alzheimer’s, Parkinson’s, and asthma [9–14]. Higher levels of this protein are generally associated with a poorer prognosis for patients, including overall survival of cancer patients. CHI3L1 is involved in cancer progression by promoting angiogenesis, proliferation, and migration of cancer cells and modulating the immunologic background of carcinogenesis. Therefore, CHI3L1 protein is a target for precision medicine, and recent studies show a broad anti-cancer spectrum of CHI3L1 inhibition [15–17].

The presence of the BBB can often lead to impaired drug administration due to missed targets. Overcoming the BBB is a critical step in the early stages of research to discover new targeted drugs for glioblastoma. One of the latest proposed strategies is the use of new formulations or carriers to improve drug delivery, such as EGFR inhibitors in liposomes or polymers, which are currently being tested in clinical trials [18–20]. One of the challenges in the initial delivery of drugs against GBM is the relevance of in vitro models. Undoubtedly, 3D spheroid models are considered more physiologic compared to traditional two-dimensional cell cultures [21]. Moreover, it is now clear that the tumor microenvironment supports carcinogenesis. Therefore, multicellular spheroid models should be considered as a more relevant model for drug discovery [22].

Physical analysis is a unique technique that enables qualitative analysis of whole spheroids in drug discovery, in terms of compound penetration into tumor models [23, 24]. To overcome the technical limitations of the spheroid analysis, we measured physical parameters indicative of targeted drug uptake in this study. In this article, we present the penetration of G721-0282 compound into GBM spheroids analyzed using a physical cytometer and a label-free method.

This article analyzes for the first time the effects of G721-0282, a CHI3L1 inhibitor, on the expression of proteins involved in intercellular junctions, such as E-cadherin, N-cadherin and VCAM-1, in GBM spheroids composed of glioblastoma U-87 MG cells, macrophages, and HMEC-1 endothelial cells. Our team previously described the broad spectrum of CHI3L1 inhibition by G721-0282 in GBM spheroids, especially concerning its antiangiogenic effect [15]. Park et al. also demonstrated the anti-cancer effect of this inhibition on osteosarcoma MG-63 cells [17]. In this article, we also present an analysis of CHI3L1 and inhibitor interactions provided in silico and also with ATR-FTIR spectroscopy.

## Materials and methods

### Docking

The crystal structure of human CHI3L1 (PBD:1NWR [25]) was obtained from the Protein Data Bank (http://www.rcsb.org). All ligands and water molecules were removed and then polar hydrogen atoms and Kollman charges were added to the protein structure using AutoDock Tools 1.5.6 [26]. The structure of G721-0282 was optimized using the DFT functionality with the basis set B3LYP/6–31 + G (d.p) [27–29]. The calculations were performed using the Gaussian 2016 C.01 software package [30]. To prepare the G721-0282 ligand, partial charges were calculated and nonpolar hydrogens were merged and rotatable bonds were assigned. The values for depletion were set to 8, 16, 24, and 60. The size of the lattice box was adjusted to include the entire monomer. Interactions were further analyzed using Discovery Studio Visualizer v.20 (https://www.3ds.com/). Visualizations were performed using ChimeraX 1.4 software [31] and LigPlot + v.2.2 software [32].

The molecular dynamic (MD) simulation was carried out with the software Gromacs 2021.2 [33] using the force field Charmm36m [34]. The topology and force field parameters were generated with the CHARMM-GUI server [35, 36]. The TIP3P water model was used for the solvation of the system. KCl was used for the electrical neutralization (the concentration was set to 0.15 M). The cut-off was set to 1.2 nm for the long-range van der Waals and electrostatic interaction. The MD simulation was performed for a period of 100 ns with a step of 2 fs, considering a constant pressure of 1 bar and a constant temperature of 303 K. The free binding energy ΔGbind was calculated using the tool gmx_MMPBSA v.1.6.1 [37].

### Infrared spectroscopy

ATR-FTIR spectra were recorded using a Nicolet 6700 FTIR spectrometer (Thermo Scientific, USA) equipped with a Golden Gate Mk II ATR accessory with a heated diamond plate (PIKE Technologies) continuously purged with dry air. An aliquot of 10 µl of the sample was pipetted onto the diamond surface and allowed to dry. All spectra were recorded with an average of 512 scans with a resolution of 4 cm − 1 in a spectral range of 4000–400 cm − 1 at a constant temperature of 22ºC (71.60 F).

Spectral analysis was performed using OriginPro, version 2023b (OriginLab Corporation, Northampton, MA, USA). Spectral preprocessing included automatic atmospheric correction, baseline subtraction, and smoothing with the Savitzky–Golay filter (polynomial order 2, window size 19). The spectra were normalized to the ν(CH2) band at ~ 2920 cm-1.

### Cell lines

U -87 MG glioblastoma cells (ATCC, American Type Culture Collection ATCC, Old Town Manassas, VA, USA) were cultured in DMEM containing 4.5 g/L glucose, while THP-1 monocytes (ATCC) were cultured in RPMI-1640 (Gibco, ThermoFischer Scientific, Wilmington, DE, USA) supplemented with 50µM β-mercaptoethanol (Sigma-Aldrich, St. Louis, MO, USA). The medium was supplemented with 10% FBS (Sigma-Aldrich) and 1% penicillin-streptomycin solution (Sigma-Aldrich) of the final volume. HMEC-1 human microvascular endothelial cells (ATCC) were cultured in MCDB131 medium supplemented with 10 mM L-glutamine, 10 ng/mL FGF (cat. PHG0311L, ThermoFisher Scientific) and 1 µg/mL hydrocortisone (cat. H0888, Sigma-Aldrich). Cells were passaged with TrypLe (Gibco) for adherent cell lines, and confluence was not allowed to exceed 70%. The media were changed twice a week. Cells were cultured in an atmosphere of 5% CO_2_ and 95% humidity in a HeraCell 150i incubator (ThermoFischer Scientific).

### Monocyte differentiation

THP-1 monocytes were differentiated into macrophages by incubating them for 24 h in a complete RPMI-1640 medium (Gibco) with the addition of 100nM PMA (cat. P1585; Sigma-Aldrich) [37].

### Spheroids formation

Spheroids were formed using U-87 MG glioblastoma cells, HMEC-1 endothelial cells, and macrophages as previously described [15]. The 96-well plate (3D PrimeSurface^®^ 96 V, cat. MS-9096VZ, Akita Sunitomo Bakelite, Akita, Japan) was used to ensure uniform spheroid formation for biophysical analyzes. For protein and mRNA expression analysis, spheroids were cultured in low adhesion flasks for one month and treated with G721-0282 at a specified concentration for 72 h, and the next one 72 h with fresh inhibitor concentrations (2 × 72 h). The drug was dissolved in a complete medium containing 0.1% DMSO (cat. D8418, Sigma-Aldrich), and a solvent control was used. The spheroids were fixed according to the described procedure [15, 38] and embedded in paraffin.

### Biophysical parameter of spheroids

The W8 Physical Cytometer (Cell Dynamics, Bologna, Italy) [23, 24] was used as a label-free and non-invasive method to determine the biophysical parameters of spheroids, including mass density, weight, and diameter. Spheroids were cultured for four days to ensure that their diameter did not exceed 600 μm and were then treated with G721-0282 for 2 × 72 h before analysis. The study included four groups of spheroids: those treated with 100 μm G721-0282, those treated with 50 μm G721-0282, those treated with complete medium with DMSO (0.1%) used to dissolve the compound (solvent control), and those treated with complete medium. The experiment was performed in triplicate.

### Scanning electron microscopy

Label-free and non-invasive methods were used to image spheroids (previously fixed in 4% PFA) using scanning electron microscopy (SEM) under low-pressure conditions and environmental scanning electron microscopy (ESEM). The SEM and ESEM analysis was performed at 50–70% humidity and − 4 °C using the Quanta 3D 200i microscope (FEI Company, Hillsboro, OR, USA).

### Immunohistochemical reactions

The immunohistochemical reactions were carried out on 4 μm sections of spheroids. Paraffin removal and antigen retrieval were performed in PT-Link (Dako, Glostrup, Denmark) using EnVision FLEX Target Retrieval Solution (97 °C, 20 min, pH 6.0 (cat. K8005) for CD31 and pH 9.0 (cat. K8004) for CHI3L1, N-cadherin, E-cadherin, VE-cadherin, VCAM-1, EGFR and IBA1). 1% BSA (bovine serum albumin) was used as a blocker, followed by the blocking of endogenous peroxidase with EnVision FLEX Peroxidase Blocking Reagent (Dako) (RT, 5 min). As blocker 1% BSA (bovine serum albumin) (RT, 30 min) was used, followed by blocking of endogenous peroxidase with EnVision FLEX Peroxidase Blocking Reagent (Dako) (RT, 5 min). Incubation with primary antibodies was performed overnight at 4 °C using the following antibodies: anti-CHI3L1 (cat. AF2599, goat polyclonal, R&D), anti-N-cadherin (cat. sc-8424, mouse monoclonal, Santa Cruz), E-cadherin (cat. sc-8226, mouse monoclonal, Santa Cruz), VE-cadherin (cat. sc-9989, mouse monoclonal, Santa Cruz) (all antibodies in dilutions 1:50), anti-VCAM-1 (cat. ab134047, 1:500, rabbit monoclonal, Abcam), anti-EGFR (cat. M3563, mouse monoclonal, 1:200, Dako), anti-CD31 (cat. ab9498, 1:500, mouse monoclonal, Abcam, Cambridge, UK) or anti-IBA1 antibody (cat. 019-19741, 1:500, rabbit polyclonal, FUJIFILM Wako, Osaka, Japan). Antibody dilutions were prepared with 1% BSA in PBS/0.1% Tween20. Reagents used for immunohistochemical reactions were part of the EnVision Flex/High pH kit (K8010, Dako). The EnVision FLEX/HRP (RTU, Dako, 1 h, RT) secondary antibody system was used for mouse and rabbit antibodies. In addition, donkey anti-goat secondary antibodies conjugated to HRP (1:400, Jackson ImmunoResearch, Suffolk, UK) were used. The slides were then incubated in EnVision Flex substrate buffer (Dako) with DAB (cat. DM827, Dako) for 10 min at room temperature. To counterstain the slides, EnVision Flex Hematoxylin Solution (cat. K8008, Dako) was used for 5 min at room temperature. Finally, the slides were covered with Dako Mounting Medium (Dako) [39].

### Western blot

Spheroids and U-87 MG samples were homogenized in RIPA buffer with EDTA, Coctail protease inhibitor (Heat™ Protease Inhibitor Coctail×100), and 0.5 mM PMSF (phenylmethanesulfonyl fluoride) (all Thermo Fischer). The NanoDrop1000 (Thermo Fischer) was used with a bicinchoninic acid assay (Pierce BCA Protein Assay Kit) to determine total protein content. Lysates were obtained by denaturing the samples in a buffer consisting of 250 mM TRIS pH 6.8, 40% glycerol, 20% (v/v) β-mercaptoethanol, 0.33 mg/ml bromophenol blue, 8% sodium dodecyl sulfate (SDS) for 5 min at 95 °C. Samples were then loaded onto a 10% polyacrylamide gel in a Mini Protean 3 device (Bio-Rad, Hercules, CA, USA) with 20 µg protein per lane according to the Laemmli method [40]. Protein transfer was performed for 1 h at 140 V in Tris-glycine buffer containing 20% methanol and 0.05% SDS, with PVDF membrane 0.45 μm (polyvinylidene difluoride) (Immobilon^®^, Millipore, Bedford, MA, USA). The blocker used was 5% milk in 0.05% TBST. Incubation was performed overnight at 4ºC with primary antibodies against CHI3L1 (cat. AF2599, goat polyclonal, 1:1000, R&D), VCAM-1 (cat. ab134047, rabbit monoclonal, 1:1000 in 5% skim milk in 0.05% TBST, Abcam), N-cadherin (cat. sc-8424, mouse monoclonal, 1:1000 in 5% skim milk in 0.05% TBST, Santa Cruz, Dallas, TX, USA), E-cadherin (cat. sc-8226, monoclonal mouse, 1:1000 in 5% skim milk in 0.05% TBST, Santa Cruz), VE-cadherin (cat. sc-9989, monoclonal mouse, 1:1000 in 5% skim milk in 0.05% TBST, Santa Cruz), β-tubulin (cat. ab6046, rabbit polyclonal, 1:1000, in 0. 1% BSA in 0.1% TBST, Abcam), GAPDH (cat. 2118, rabbit monoclonal, 1:1000, in 5% milk in 0.05% TBST, Cell Signaling, Denver, MA, USA). Incubation with secondary antibodies conjugated to horseradish peroxidase (HRP) was performed for 1 h RT with donkey anti-rabbit antibody (1:6000, in 5% milk in 0.05% TBST, Jackson ImmunoResearch, Suffolk, UK), donkey anti-mouse (1:3000, in 5% milk in 0.05% TBST, Jackson ImmunoResearch) or donkey anti-goat antibody (1:10000, in 5% milk in 0.05% TBST, Jackson ImmunoResearch). Chemiluminescence reactions were detected with Luminata Forte Immobilon^®^ Western HRP substrate (Thermo Fischer) and visualized with the ChemiDoc TM MP system (Bio-Rad) and ImageLab software (Bio-Rad) [41, 42].

### The Droplet Digital PCR™ (ddPCR)

The absolute number of mRNA copies of the genes in the analyzed materials was determined using the Droplet Digital PCR method. The RNeasy Mini Kit (cat. 74104, Qiagen, Hilden, Germany) was used to isolate total RNA. For reverse transcription (RT-PCR), the iScript™ Reverse Transcription Supermix for RT-qPCR (cat. 1708840, Bio-Rad, Hercules, CA, USA) was used; 35 ng of RNA from each sample was reverse-transcribed using the C1000 Touch Thermal Cycler (Bio-Rad). In the experiment, the reaction conditions were primed for 5 min at 25 °C, reverse transcription for 20 min at 46 °C, and final inactivation of the reverse transcriptase for 1 min at 95 °C. The ddPCR reaction mixes contained 2.5 µl of RT product, 1 µl of TaqMan-specific probe (Applied Biosystems, Foster City, CA, USA), 7.67 µl of molecular biology grade water, and 10 µl of 2X ddPCR™ MasterMix for Probes (Bio-Rad). The TaqMan-specific primers were used to evaluate the mRNA expression of the analyzed genes: Hs01023895_m1 (CDH1), Hs00983056_m1 (CDH2), Hs00901465_m1 (CDH5), Hs01003372_m1 (VCAM-1). The reaction mixtures (20 µl) were loaded into a cartridge (Bio-Rad) and mixed with 50 µl Droplet Generation Oil for Probes (Bio-Rad) in the QX100 Droplet Generator (Bio-Rad). The droplets obtained from each sample were then transferred to a 96-well PCR plate (Eppendorf, Hamburg, Germany). The reaction conditions: Enzyme activation for 10 min at 95 °C, followed by 40 cycles of denaturation (30 s, 94 °C) and annealing/extension (1 min, 60 °C), final enzyme deactivation for 10 min at 98 °C and final 10 min at room temperature (RT) were performed for PCR amplifications in a C1000 Touch Thermal Cycler (Bio-Rad). The plates were read automatically with the Droplet Reader (Bio-Rad). The number of positive counts per plate using the Poisson distribution was used to calculate the absolute quantification of each mRNA. The number of copies/µl (AQ) in the PCR reaction mixture represents the quantification of the target mRNA [42, 43].

## Statistical analysis

The normality of the variable distribution was analyzed using the Shapiro-Wilk test. One-way ANOVA with Tukey’s post-hoc test for multiple comparisons and Spearman’s correlation were applied. Statistical significance was defined as *p* < 0.05. Statistical analysis was performed using Statistica 13.3 (Tibco, Palo Alto, CA, USA) and Prism 9.4.1 (GraphPad, La Jolla, CA, USA) software.

## Results

### G721-0282 binds to CHI3L1 in docking studies

**Fig. 1:**
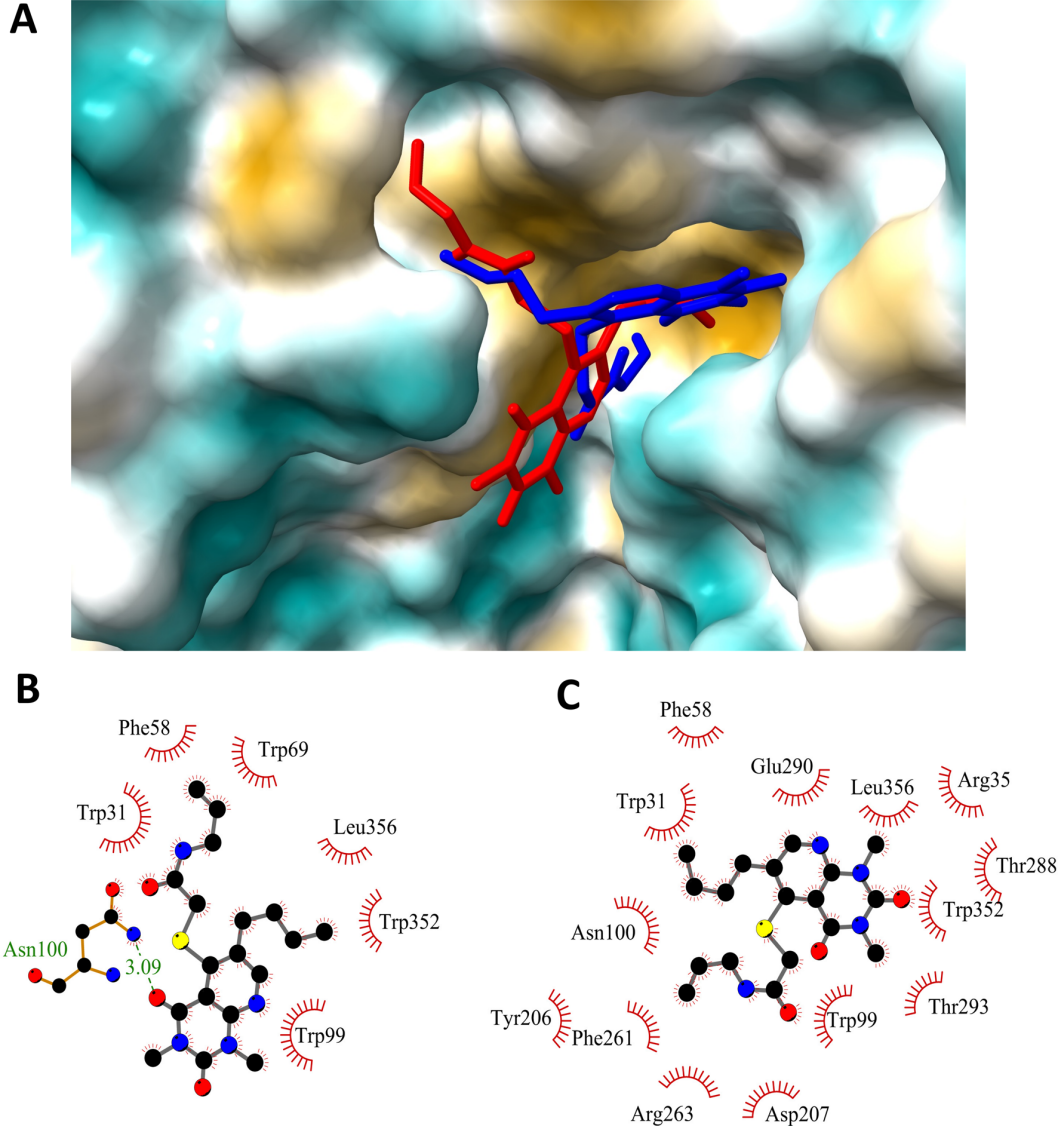
Interactions of compounds CHI3L1 and G721-0282 (the CHI3L1 inhibitor) in docking studies. Position of compound G721-0282 in the binding site of CHI3L1: **A**) the pose of two modes of compound G721-0282 (mode 1 red, mode 2 blue) in complex with CHI3L1; **B**) the 2D representation of the interactions of mode 1; **C**) 2D diagram of the interactions of mode 2. The crystal structure of human CHI3L1 (PBD:1NWR, Protein Data Bank); protein structure adjustment: using AutoDock Tools 1.5.6; G721-0282 structure optimization: DFT functionality; basis set B3LYP/6–31 + G (d.p); visualizations: ChimeraX 1.4 software and LigPlot + v.2.2 software

Molecular docking studies were performed to determine how G721-0282 interacts with CHI3L1. The crystal structure of CHI3L1, PBD: 1NWR [44] was used for the calculations. The docking results showed that the binding affinity is negative. This indicates that a stable complex is formed. For the best pose, the binding affinity is negative, − 7.81 kcal/mol. The distribution of the interaction energy was calculated as follows: −11.28 kcal/mol for the sum of van der Waals energy, hydrogen bonding energy, and desolvation-free energy and − 0.44 kcal/mol for the electrostatic energy. Figure 1A shows the position of compound G721-0282 in the binding site of CHI3L1 for mode 1 (red) and mode 2 (blue) and the 2D representation of the interactions between ligand and receptor (Fig. 1B and C). In mode 1, a hydrogen bond is formed between an oxygen substituent in the ring and the ASN100 residue (3.08Å). The butyl chain interacts with PHE261, TRP352, and TRP99 via π-alkyl contacts and with LEU356 via an alkyl contact. The N-allyl unit is surrounded by the residues TRP31, TRP69, PHE58, and SER30 and interacts via π-alkyl contacts. The pyrido[2,3-d]pyrimidine ring is involved in the π-π stacking interactions with TRP99 and the π anion with GLU290. The molecular docking results also showed that the differences in binding affinity between the best position (mode 1, Fig. 1B) and subsequent modes are small. The binding affinity of mode 2 is only 0.30 kcal/mol lower, i.e. −7.50 kcal/mol. The distribution of the interaction energy was determined as follows: −10.37 kcal/mol for the sum of van der Waals energy, hydrogen bonding energy, and desolvation-free energy, and − 0.43 kcal/mol for the electrostatic energy.

The position of mode 2 in the CHI3L1 site is shown in Fig. 1A. The 2D representation of the interactions is shown in Fig. 1C. The components of the ligand are positioned slightly differently at the binding site. Hydrogen bonds were not found. The hydrophobic interactions are mostly the same residues but with different groups of the mode 2 molecule. The butyl chain interacts with ASN100, TRP31, PHE58, and TRP99 via π-alkyl contacts. The N-allyl moiety is surrounded by the residues TYR206, TRP99, PHE261, TRP352, ARG263, and ASP207 and interacts via π-alkyl contacts. The pyrido[2,3-d]pyrimidine ring is involved in the π-cation interactions with ARG35, the π-anion with GLU290, and interacts via π-alkyl contacts with TRP31, THR288, LEU356, THR293.

**Fig. 2:**
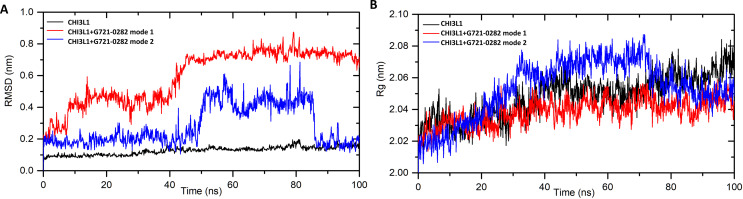
Molecular dynamics simulations for compounds CHI3L1 and G721-0282 (the CHI3L1 inhibitor) complexes. The plots of root mean square deviation (RMSD) - **A**) and radius of gyrations (Rg)– **B**) for CHI3L1 and the complexes with G721-0282, mode 1 and mode 2, during the MD 100 ns simulation. The molecular dynamic provided with Gromacs 2021.2 software; topology and force field parameters generated with the CHARMM-GUI server; free binding energy ΔGbind: calculated using the tool gmx_MMPBSA v.1.6.1

Molecular dynamics simulations for CHI3L1 and CHI3L1- G721-0282 complexes for mode 1 and mode 2 were performed to confirm the stability of the complexes and to calculate the free enthalpy of binding. The simulation was performed for 100 ns. The results were analyzed using parameters such as root mean square deviation (RMSD) and radius of gyration (Rg). The RMSD plots (indicating structural changes from the initial coordinates) of the protein backbone with and without ligands are shown in Fig. 2A. The analysis shows that the RMSD for the complex with G721-0282 mode 1 increased from 0 to 50 ns and then reached stabilization with a value of 0.73 ± 0.04 nm. For the complex with mode 2, after a phase of stabilization (up to 50 ns with RMSD 0.20 ± 0.03 nm), there is a rapid increase of about 0.3 nm (with RMSD 0.45 ± 0.03 nm, 50–85 ns). Then, after some fluctuations, the RMSD decreased again to a value of 0.18 ± 0.04 nm. The higher value of the complexes than CHI3L1 (0.14 ± 0.04 nm) indicates a structural change in the binding of G721-0282. For both mode 1 and mode 2 complexes, the RMSD values are low, indicating the formation of stable complexes. However, stabilization is achieved faster for mode 1. The compactness of the protein was analyzed by plotting the Rg values against time. The graph is shown in Fig. 2B. In the initial state, the Rg value of the free CHI3L1 and CHI3L1– G721-0282 complex was 2.03 nm, 2.02 nm (mode 1), and 2.03 nm (mode 2), respectively. During the simulation period, Rg increased only slightly for the complex with mode (1) The results indicate very negligible atomic fluctuations and the complex remained compact and stable during the simulation period. Slightly larger fluctuations were observed for the mode 2 complex. Next, the binding free energy was calculated from the trajectories of the MD complex using the gmx_MMPBSA tool [45]. The average ΔGbind was found to be − 17.87 ± 2.88 kcal/mol and − 16.65 ± 2.75 kcal/mol for the CHI3L1–G721-0282 (mode 1) and CHI3L1–G721-0282 (mode 2) systems, respectively. The contribution of the interaction energy was determined as follows: Van der Waals − 36.04 ± 1.09 and − 33.68 ± 0.64, electrostatic − 2.40 ± 0.32 and − 3.53 ± 0.21 kcal/mol, for modes 1 and 2, respectively. The strongest van der Waals interactions are observed with Trp99 (− 4.96 kcal/mol), Trp31 (− 3.07 kcal/mol), Trp69 (− 1.44 kcal/mol), Asn100 (− 1.41 kcal/mol), Trp352 (− 1.34 kcal/mol) for mode 1 and Trp99 (− 3.61 kcal/mol), Trp31 (− 2.23 kcal/mol), Trp352 (− 1.84 kcal/mol), Phe261 (− 1.36 kcal/mol), Asn100 (− 1.32 kcal/mol), Thr288 (− 1.09 kcal/mol) for mode (2) The higher value (more negative) for the system with mode 1 indicates that the probability of G721-0282 interacting with CHI3L1 as in mode 1 is higher than in mode 2.

### Infrared spectroscopy confirms the complex between CHI3L1 and G721-0282

**Fig. 3:**
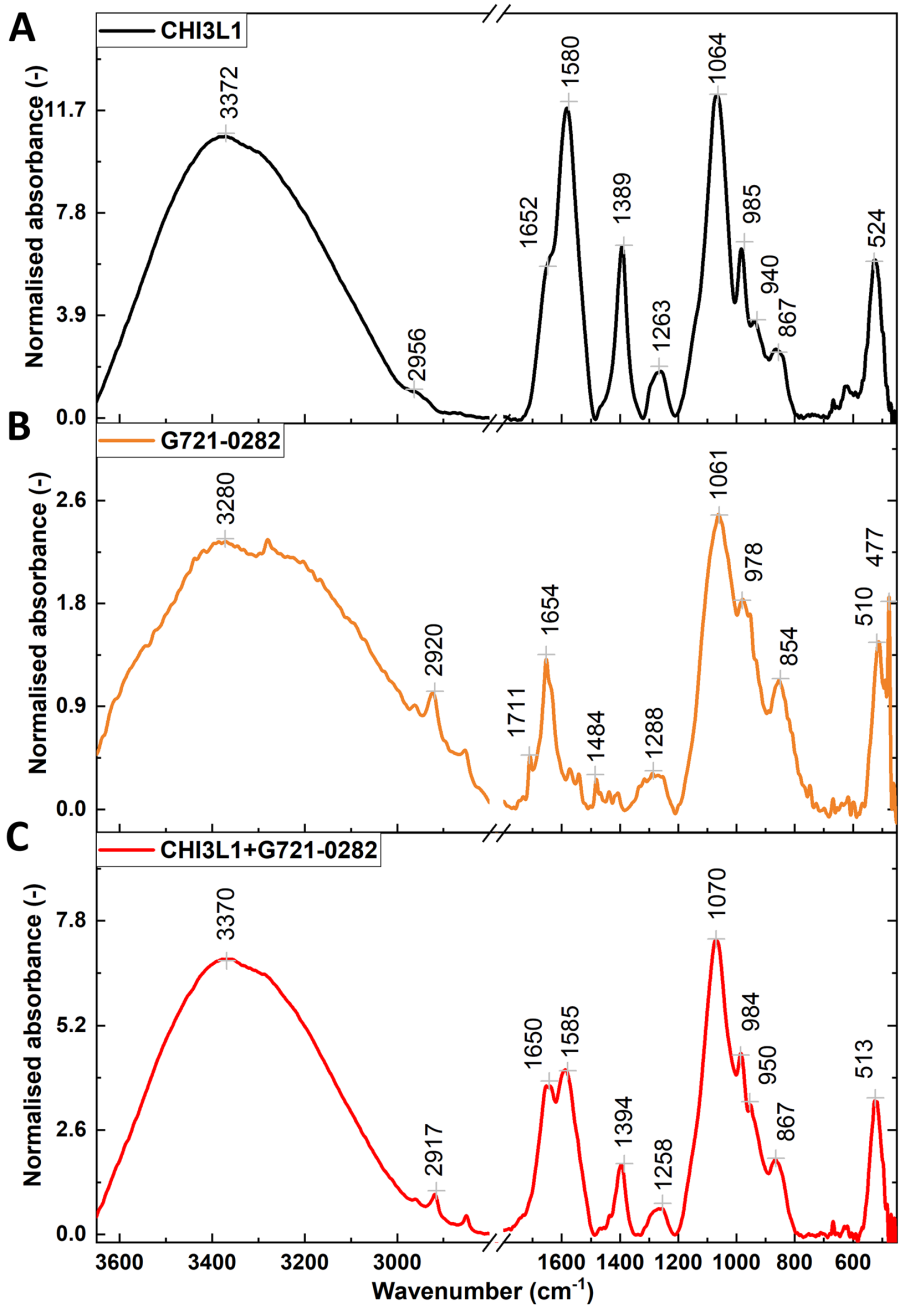
The complex between compounds CHI3L1 and G721-0282 (the CHI3L1 inhibitor) in infrared spectroscopy. Normalized ATR-FTIR spectra of CHI3L1 protein (**A**), G721-0282 (**B**), and CHI3L1 in the presence of inhibitor (**C**). Spectra recorded: Nicolet 6700 FTIR spectrometer (Thermo Scientific); spectral analysis: OriginPro, version 2023b (OriginLab Corporation) and preprocessing: Savitzky–Golay filter (polynomial order 2, window size 19). The spectra were normalized to the ν(CH2) band at ~ 2920 cm-1; ATR-FTIR: Fourier transform infrared spectroscopy

Attenuated total reflectance Fourier transform infrared spectroscopy (ATR-FTIR) was used to investigate the effect of the inhibitor G721-0282 on the CHI3L1 protein. This vibrational spectroscopy technique is widely used to study proteins [46] including glycoproteins [47] and protein-inhibitor interactions [48]. The sensitivity of FTIR spectroscopy to changes in the secondary structure of proteins allows precise monitoring of conformational changes upon inhibitor binding. This property makes FTIR spectroscopy an invaluable tool for detecting subtle shifts in protein structure that are critical for understanding mechanistic interactions at the molecular level. Figure 3 shows the spectra obtained for the CHI3L1 protein in PBS buffer (Fig. 3A), the compound G721-0282 (100 µM) in PBS (Fig. 3B) and the glycoprotein in the presence of a 5:1 inhibitor (Fig. 3C). In the range 3600–2900 cm-1 a broad and strong band can be observed, which is due to the overlap of the stretching vibrations of OH groups in water and NH groups in proteins. Weak bands between 2900–2800 cm-1 originate from CH stretching of methyl and methylene groups. While these bands are insensitive to protein conformation, the positions of the maxima of the amide bands, which correspond to the vibrations of the peptide binding groups in the 1700–1500 cm-¹ range, are strongly influenced by changes in the hydrogen bonding network. A shift of the amide I band from 1652 cm-¹ to 1650 cm-¹ was observed after the formation of a complex between the CHI3L1 protein and the compound G721-0282. This downward shift is attributed to the development of a new hydrogen bond between the protein and the inhibitor, indicating a possible change in the secondary structure of the protein. In addition, the amide II band, which originates from NH2 deformation, is shifted upward after binding of the inhibitor. In the ATR-FTIR spectra of the inhibitor, an intense band is observed in the discussed region, mainly originating from the C = O and C = C vibrations at about 1654 cm-¹. The spectral range of wavenumbers between 1200 and 800 cm-¹ is used to identify the stretching vibrations of C–O and C–C groups in proteins and inhibitors. However, this range can be difficult to analyze due to the overlapping absorption of phosphate and sulfate groups in solvents. In addition, the ATR-FTIR spectroscopic analysis results showed significant changes in the vibrational spectrum of the protein-inhibitor complex, indicating specific molecular interactions. The most notable changes apply to the Amide bands. For example, the Amide I band shifts by 2 cm⁻¹ and the Amide II by 5 cm⁻ towards lower wavenumbers when the complex was formed between the CHI3L1 protein and compound G721-0282. This shift indicates that new hydrogen bonds were formed, probably resulting from the interaction between the C = O group of the inhibitor and the NH2 group of an aspartic amino acid residue of the protein. This shift could also indicate a change in the secondary structure of the protein. In addition, there was an upward shift of the amide II band after the binding of the inhibitor, further supporting the direct interaction hypothesis.

### G721-0282 penetrates GBM spheroids

**Fig. 4:**
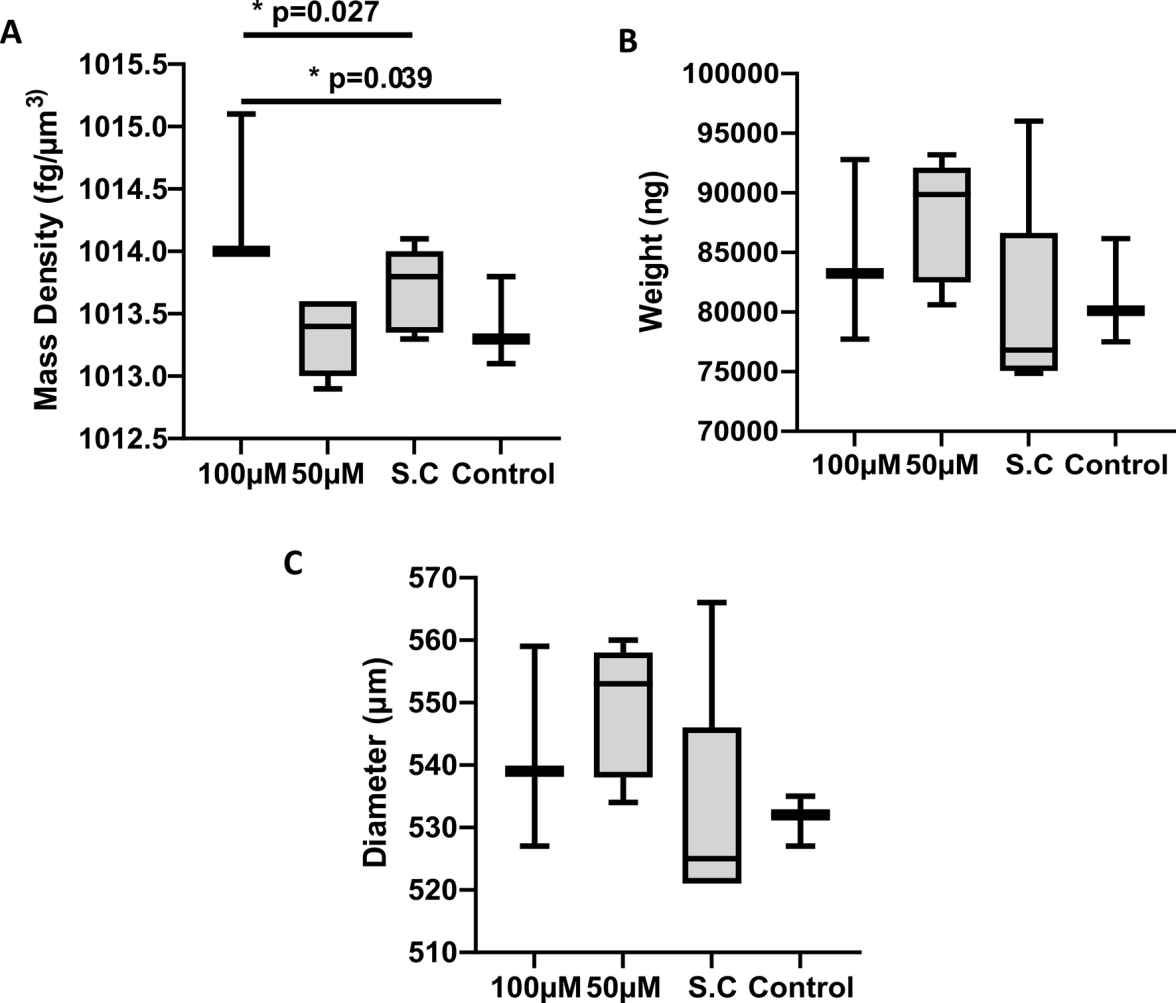
Mass density, weight, and diameter of spheroids following G721-0282 treatment. Spheroids consisting of U-87 MG glioblastoma cells, HMEC-1 endothelial cells, and macrophages were analyzed with W8 Physical Cytometer (Cell Dynamics) after incubation with 100 and 50µM of G721-0282; SC: solvent control: spheroids cultured with medium with addition of 0.1% DMSO (the solvent for G721-0282) occurred at a concentration of 100 mM of G721-0282; control: spheroids in culture medium without inhibitor. One-way ANOVA followed by Tukey’s post-hoc test, with a significance level of *p* < 0.05

Biophysical parameters of spheroids are present in Fig. 4. One-way ANOVA revealed that G721-0282 significantly affected spheroid mass density (*F*_3,12_ = 5.19, *p* = 0.016) (Fig. 4A), but had no effect on their weight and diameter (*F*_3,12_=1.17, *p* = 0.361; *F*_3,12_ =1.52, *p* = 0.260, respectively) (Fig. 4B and C). Post hoc comparison using Tukey’s test showed that that the spheroids treated with 100 µM G721-0282 had a significantly higher mass density compared to the control and spheroids treated with 50 µM. This effect shows the possibility of the uptake of CHI3L1 inhibitor (G721-0282 compound) into spheroids. Additionally, the above effects confirmed the homogeneity of the spheroid population used in the experiment since, as mentioned above, they did not differ in terms of weight in and diameter. No cytotoxic effect of G721-0282 was observed, as indicated by no change in spheroid diameter in all groups.

### Scanning electron microscopy shows no changes in GBM spheroids after treatment with G721-0282

**Fig. 5:**
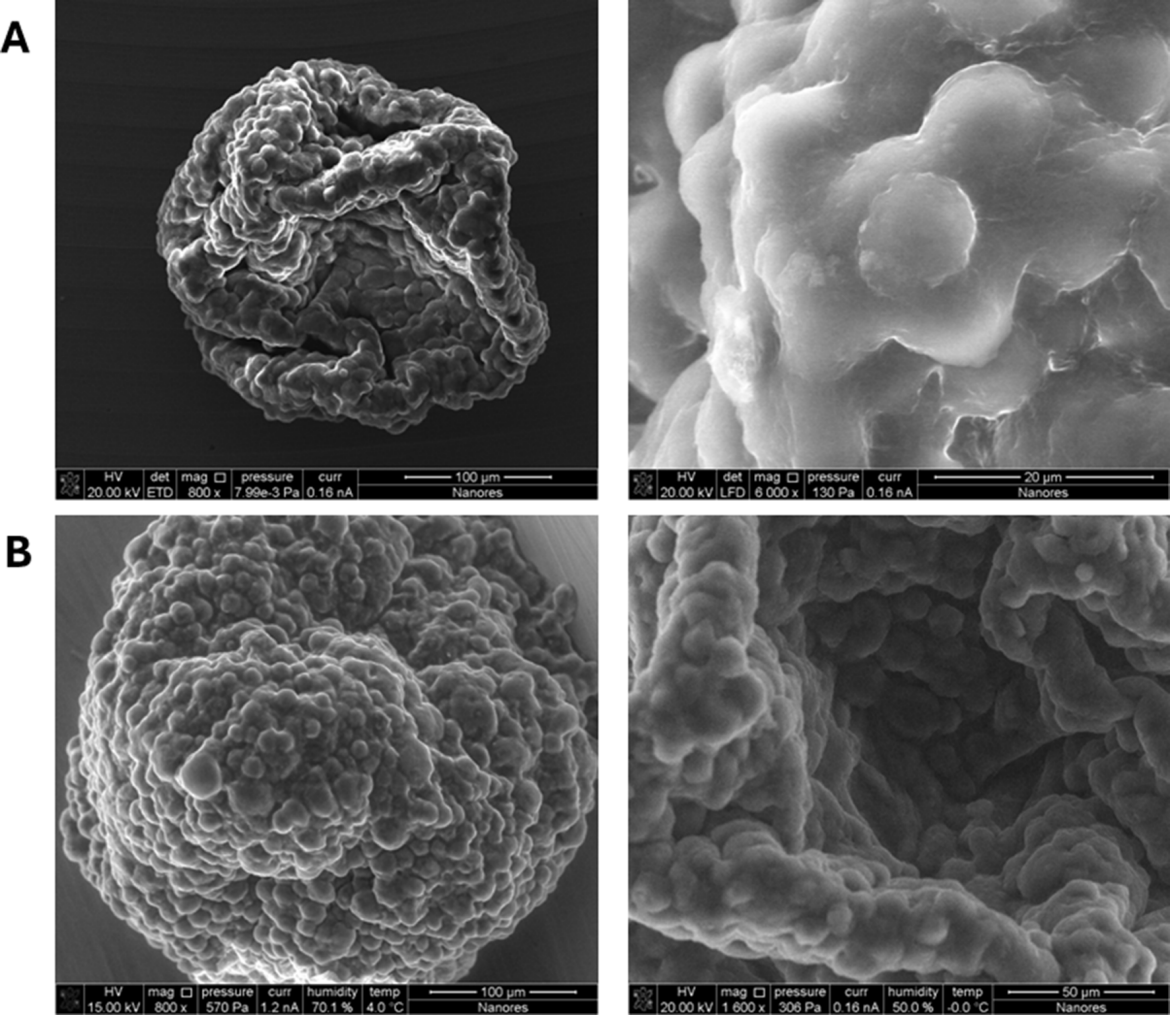
Label-free visualization of spheroids using a scanning electron microscope. Spheroids, consisted of U-87 MG glioblastoma cells, HMEC-1 endothelial cells and macrophages, undergo observation in low pressure in SEM (scanning electron microscope) (**A**) and also in environmental SEM (ESEM) (**B**); images performed using Quanta 3D 200i microscope (FEI Company); magn. 800-6000x

Spheroids analyzed in the W8 Physical Cytometer (Cell Dynamics) were next visualized by SEM in low pressure condition and ESEM under environmental conditions, and the representative images are shown in Fig. 5. This technique avoids the fixation procedures typical of ESEM and allows the observation of the surface structure of the spheroids. At this level, no differences were observed between the spheroids in all treated groups. The spheroids were characterized by a high homogeneity in terms of weight and diameter, but had different shapes (Fig. 5).

### Immunohistochemical reactions show changes in cadherins and VCAM-1 after treatment with G721-0282

**Fig. 6:**
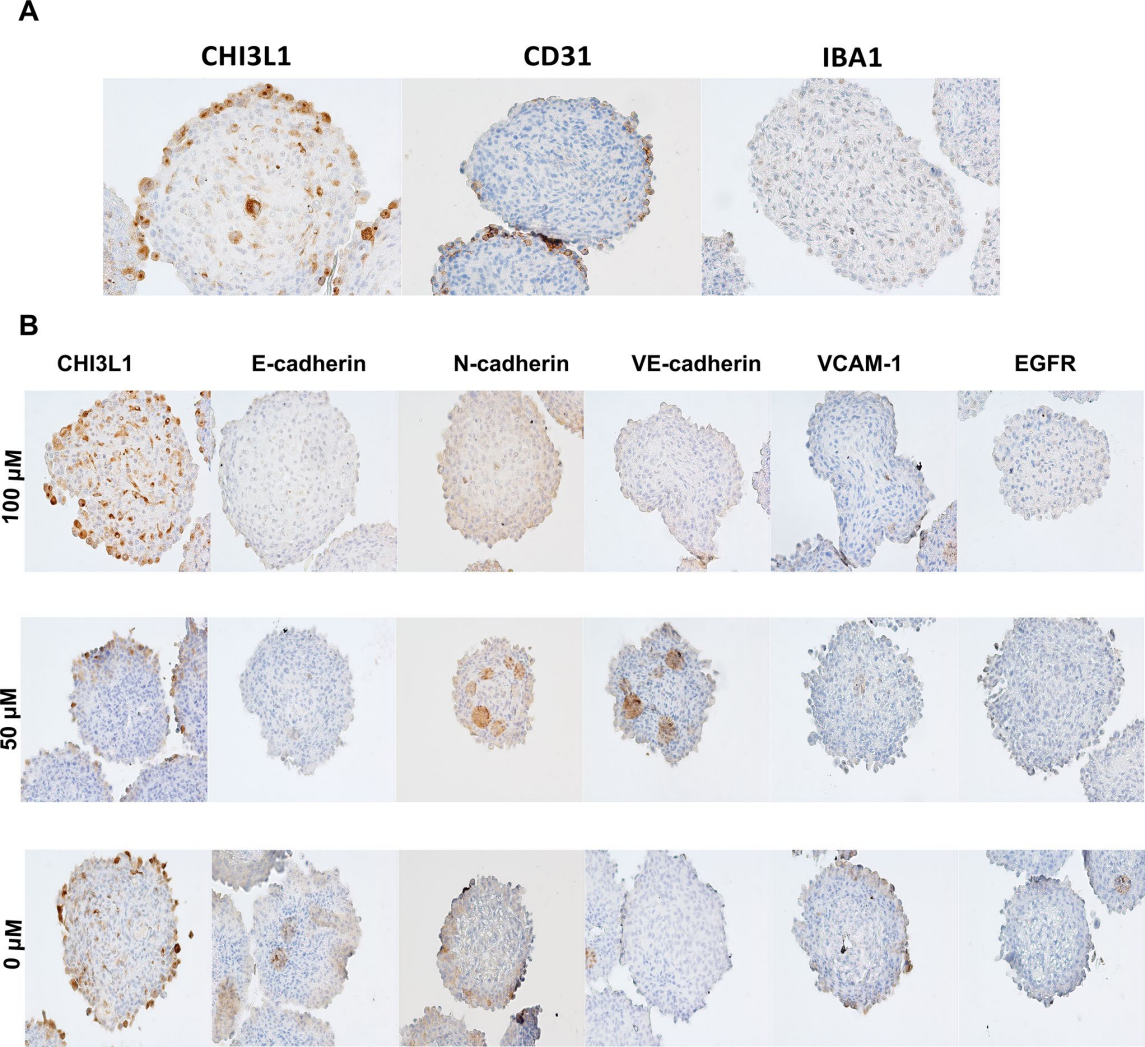
Immunohistochemical images of spheroids. Typical markers expression were analyzed using immunohistochemical reactions in U-87 MG glioblastoma cells to marked CHI3L1 with anti-CHI3L1 antibody (cat. AF2599, 1:50, R&D) endothelial cells HMEC-1 positive for CD31 with anti-CD31 antibody (cat. ab9498,1:500, Abcam) and macrophages expressed IBA1, localized by anti-IBA1 antibody (cat. 019-19741, 1:500, FUJIFILM Wako) to confirm three cell components of spheroids used in the study (**A**); changes in the expression level and localization of CHI3L1, cadherins, VCAM-1 and EGFR after treatment with G721-0282 compound (the inhibitor of CHI3L1) (**B**); light microscope BX41 (Olympus); magn.100x; CHI3L1: chitinase-3- like protein 1; EGFR: epidermal growth factor receptor; VCAM-1: vascular cell adhesion molecule 1

Immunohistochemical reactions were analyzed with a light microscope (BX41, Olympus, Tokyo, Japan). IHC reactions showed expression of markers characteristic of three cell types that form the spheroids: CHI3L1-positive U-87 MG glioblastoma cells, CD31-positive endothelial cells (HMEC-1) and IBA1 markers for macrophages (Fig. 6A). The results showed that the obtained spheroids were composed of all three cell types. In addition, histological examination confirmed the nature of the spheroids rather than clumps or clusters of cells. Spheroids treated with different concentrations of G721-0282 compound (the CHI3L1 inhibitor) showed the highest concentration of CHI3L1 and the lowest for N-cadherin at 100 µM of G721-0282 (Fig. 6B). The lowest CHI3L1 expression in U-87 MG cells was observed in spheroids treated with 50 µM of the inhibitor. Moreover, N- and VE-cadherin increased in spheroids treated with this same inhibitor concentration. For E-cadherin, the highest expression was observed in the control group. Similarly, the highest expression of VCAM-1 was also observed in the control group, and the lowest in spheroids after 100 µM of G721-0282. No changes were observed in the expression of EGFR (Fig. 6B). Additional immunohistochemical images were included in the supplementary file (Fig. S1).

### G721-0282 decreases N-cadherin, E-cadherin and VCAM-1 in GBM spheroids but not in U-87 MG cells

**Fig. 7:**
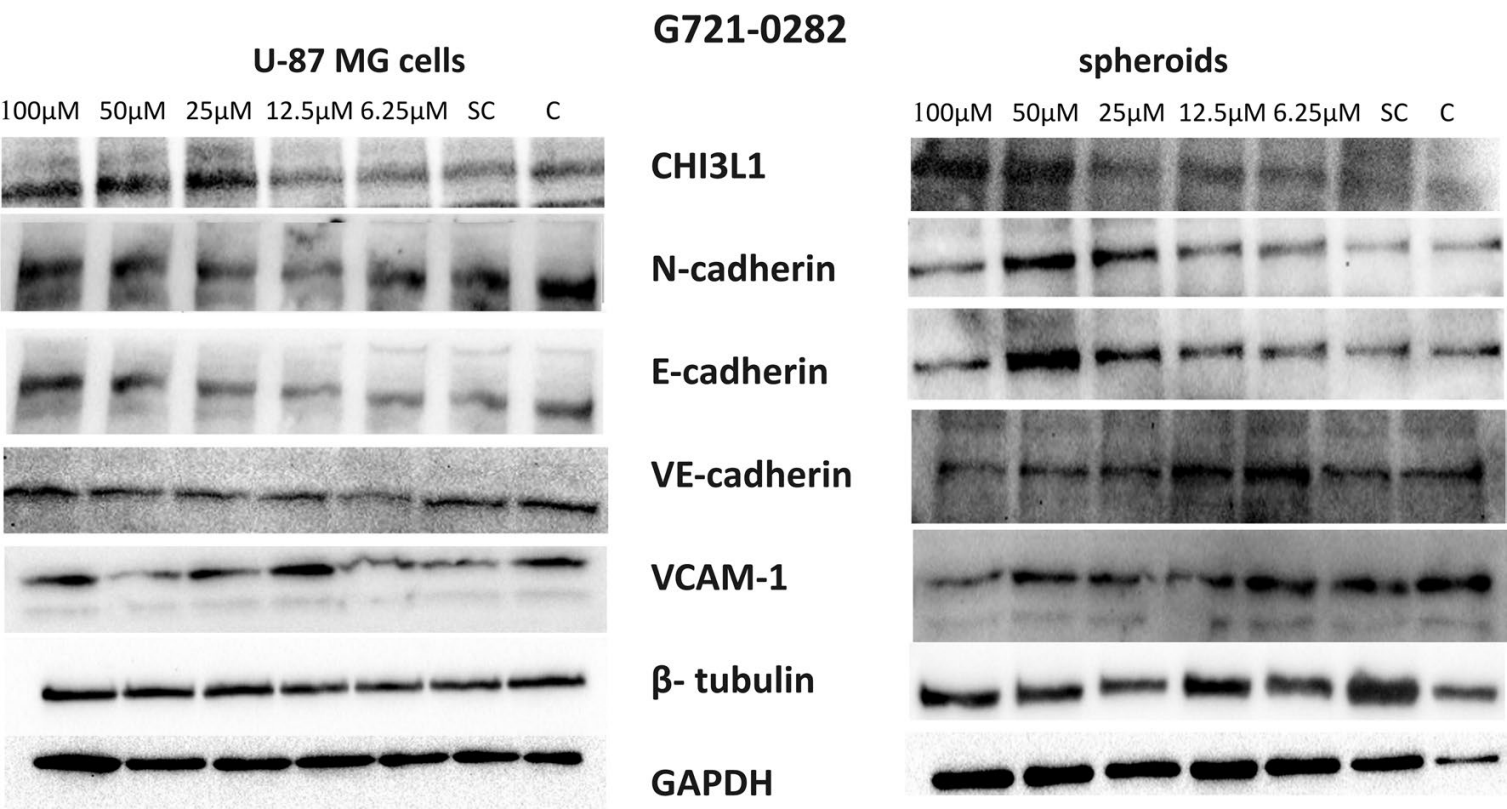
Expression of CHI3L1, N-cadherin, E-cadherin, and VCAM-1 in U-87 MG glioblastoma cells and spheroids following G721-0282 treatment using Western blot technique. Expression level after incubation with: G721-0282, a CHI3L1 inhibitor, in concentrations ranging 100 − 6.25 µM; spheroids consisted of U-87 MG glioblastoma cells, HMEC-1 endothelial cells and macrophages; SC: solvent control- cells or spheroids cultured in medium with addition of 0.1% DMSO (the solvent for G721-0282) occurred at concentration of 100 mM of G721-0282; C: control- cells or spheroids or in culture medium; β-tubulin and GAPDH were used as reference protein; CHI3L1: chitinase-3- like protein 1; VCAM-1: vascular cell adhesion molecule 1; Western blots visualization was provide with the ChemiDoc ^TM^ MP system (Bio-Rad) and ImageLab software (Bio-Rad)

Analysis of protein expression levels by Western blot methods showed an increase in the expression of CHI3L1 in U-87 MG cells and spheroids at the highest concentrations of G721-0282, which can be explained as a compensatory effect (Fig. 7). The expression of E- and N-cadherins was lowest in spheroids treated with 100 µM G721-0282. However, the highest expression level of these proteins was observed in U-87 MG cells. We also observed increased expression of E-and N-cadherins in spheroids treated with 50 µM G721-0282. Moreover, VCAM-1 levels decreased in spheroids treated with 100 µM of the CHI3L1 inhibitor, whereas no changes were observed in U-87 MG cells (Fig. 7) (full Western blots images are shown on Supplementary Fig. S2).

### G721-0282 decrease CDH2 and VCAM-1 mRNA in U-87 MG cells and in GBM spheroids, but with different results

**Fig. 8:**
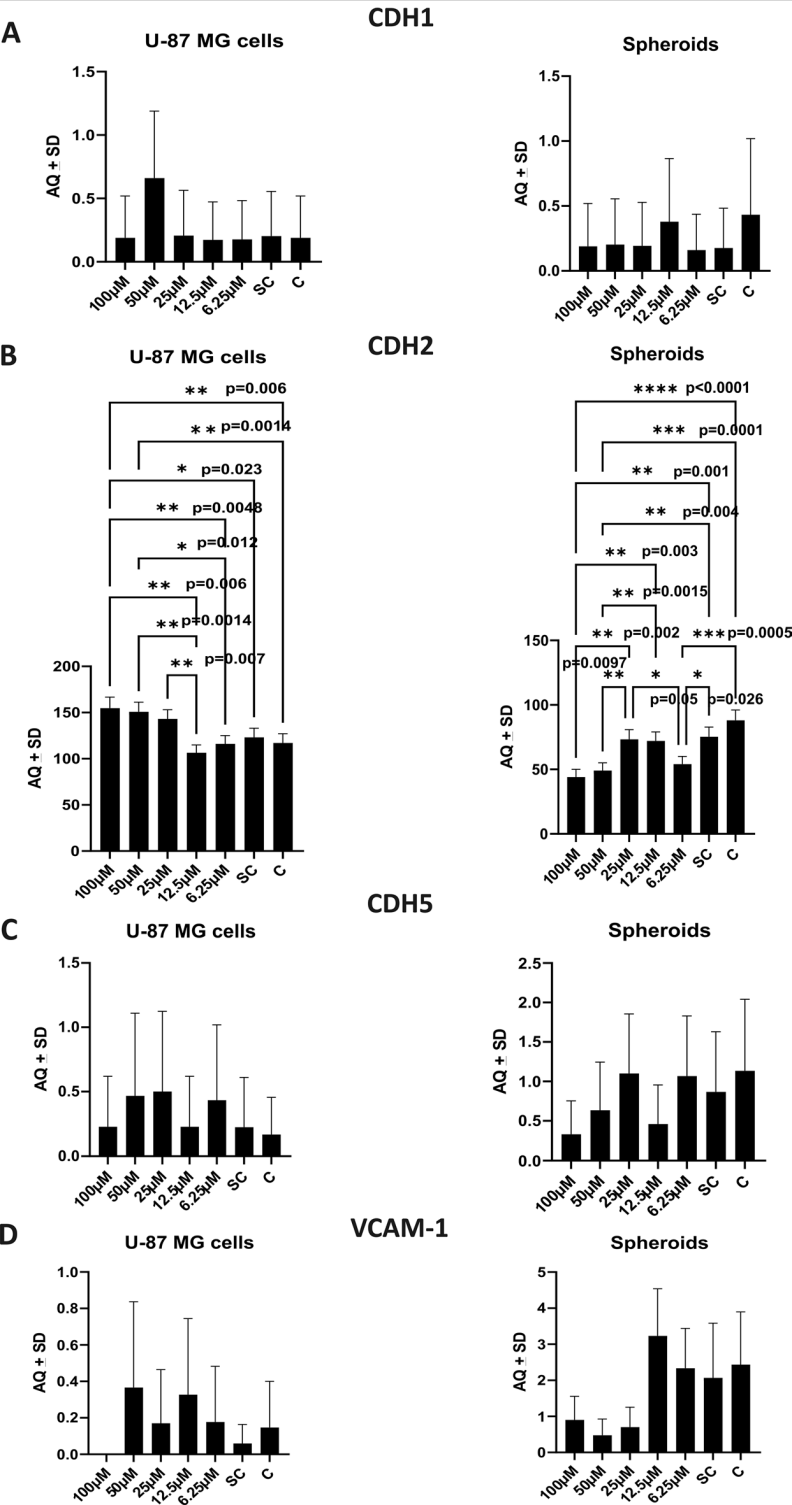
Changes in mRNA levels in U-87 MG glioblastoma cells and spheroids after G721-0282 treatment. The mRNA expression levels of (**A**) CDH1 (E-cadherin), (**B**) CDH2 (N-cadherin), (**C**) CDH5 (VE-cadherin) and (**D**) VCAM-1 in U-87 MG glioblastoma cells and also in spheroids (consisted of U-87 MG glioblastoma cells, endothelial cells HMEC-1 and macrophages) were analyzed with ddPCR technique. The TaqMan-specific primers were used to evaluate the mRNA expression of the analyzed genes: Hs01023895_m1 (CDH1), Hs00983056_m1 (CDH2), Hs00901465_m1 (CDH5), Hs01003372_m1 (VCAM-1). The QX100 Droplet Generator (Bio-Rad), C1000 Touch Thermal Cycler (Bio-Rad) and Droplet Reader (Bio-Rad) were used to obtain the number of copies/µl (AQ) in the PCR reaction mixture represents the quantification of the target mRNA; SC: solvent control- cells or spheroids cultured in medium with addition of 0.1% DMSO (the solvent for G721-0282) occurred at concentration of 100 mM of G721-0282; C: control- cells or spheroids or in culture medium; CHI3L1: chitinase-3- like protein 1; VCAM-1: vascular cell adhesion molecule 1;one-way ANOVA followed by Tukey’s post-hoc test, with a significance level of *p* < 0.05. Data from the independent Droplet Digital PCR triplicates

No statistically significant differences were observed in CDH1 (E-cadherin) mRNA level in U-87 MG and in the spheroids treated with G721-0282 by one-way ANOVA (*F*_6,14_=0.714, *p* = 0.645; *F*_6,14_=0.233, *p* = 0.958, respectively) followed by Tukey’s post-hoc test (Fig. 8A). In case of CDH2 (N-cadherin) mRNA level, significant changes were observed (one-way ANOVA) in U-87 MG (*F*_6,14_=10.75, *p* = 0.0001) and also in spheroids (*F*_6,14_=16.49, *p* = 0.0001) between groups (see Fig. 8A). Post-hoc analysis with Tukey’s test revealed, that inhibition of CHI3L1 resulted in a significant increase in CHD2 mRNA level in U-87 MG cells treated with the highest G721-0282 compound concentration compared to untreated cells or treated with lower G721-0282 concentrations (Fig. 8B). Moreover, the lowest CDH2 mRNA level was observed in cells treated with 25 µM of G721-0282 (Fig. 8B).

**Fig. 9:**
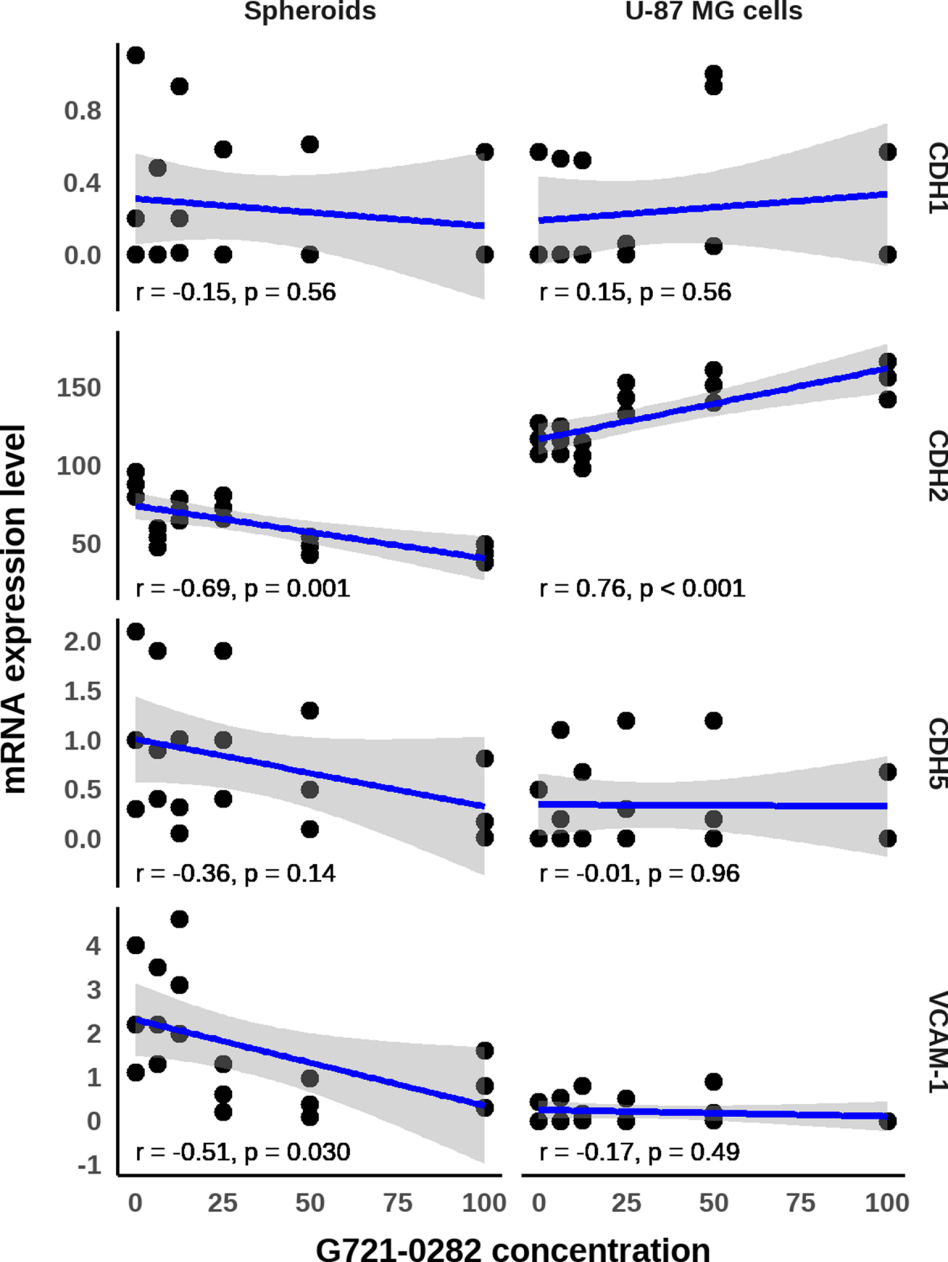
Analysis of changes in mRNA expression level in U-87 MG glioblastoma cells and spheroids after treatment with G721-0282. Data obtained from ddPCR technique for CDH1 (E-cadherin), CDH2 (N-cadherin), CDH5 (VE-cadherin) and VCAM-1 mRNA level in U-87 MG glioblastoma cells and in spheroids (consisted of U-87 MG glioblastoma cells, endothelial cells HMEC-1 and macrophages). The TaqMan-specific primers were used to evaluate the mRNA expression of the analyzed genes: Hs01023895_m1 (CDH1), Hs00983056_m1 (CDH2), Hs00901465_m1 (CDH5), Hs01003372_m1 (VCAM-1). The QX100 Droplet Generator (Bio-Rad), C1000 Touch Thermal Cycler (Bio-Rad) and Droplet Reader (Bio-Rad) were used to obtain the number of copies/µl (AQ) in the PCR reaction mixture represents the quantification of the target mRNA; SC: solvent control- cells or spheroids cultured in medium with addition of 0.1% DMSO (the solvent for G721-0282) occurred at concentration of 100 mM of G721-0282; C: control- cells or spheroids or in culture medium; CHI3L1: chitinase-3- like protein 1; VCAM-1: vascular cell adhesion molecule 1; A Spearman correlation; with a significance level of *p* < 0.05. Data from the independent Droplet Digital PCR triplicates

Conversely, in spheroids treated with the highest concentration of G721-0282 compound, a statistically significant decreased level in CHD2 mRNA levels was observed by one-way ANOVA (*F*_6,14_=16.49, *p* = 0.0001), followed by Tukey’s post-hoc test (Fig. 8B). No statistical differences were observed between spheroids cultured with 100, 50 and 6.25 µM of G721-0282. Detailed statistical differences between groups were present on Fig. 8B. The discrepancies between the effects of the G721-0282 compound on CDH2 mRNA in U-87 MG and glioblastoma spheroids may suggest a regulatory function of CHI3L1 in N-cadherin and a direct compensation effect occurred in glioblastoma cells (Fig. 8B). Furthermore, a positive and statistically significant Spearman correlation was observed between G721-0282 and an increase in CDH2 mRNA levels in U-87 MG cells (*R* = 0.76, *p* = 0.000028). In contrast, a negative strong significant Spearman correlation (*R*=-0.69, *p* = 0.0014) was observed for glioblastoma spheroids, indicating that CHI3L1 inhibition resulted in a reduction in CDH2 mRNA levels (Fig. 9). These findings suggest that the CHI3L1 inhibition lead to opposite effects in CDH2 mRNA expression in glioblastoma U-87 MG cells and glioblastoma spheroids (Fig. 9).

Analysis of results by one-way ANOVA showed that inhibition of CHI3L1 had no effect on CDH5 (VE-cadherin) mRNA level in U-87 MG cells (*F*_6,14_=0.243, *p* = 0.954) and in spheroids (*F*_6,14_=0.668, *p* = 0.677), followed by post-hoc Tukey’s test (Fig. 8C). Spearman correlations did not confirm the influence of G721-0928 on CDH5 mRNA lveelsin glioblastoma cells (*R*=-0.013, *p* = 0.96) and spheroids (*R*=-0.36, *p* = 0.14).

The inhibition of CHI3L1 resulted in a similar response from U-87 MG cells and spheroids at the level of VCAM-1 mRNA. The concentration of 100 µM of G721-0282 was found to completely inhibit VCAM-1 mRNA expression in glioblastoma cells. In spheroids, decreased of VCAM-1 mRNA were observed after treatment with compound from the range of 100 − 25 µM. However, one-way ANOVA followed by post-hoc Tukey’s test identified no statistically significant differences between the U-87 MG cell groups (*F*_6,14_= 0.561, *p* = 0.755) and the spheroid groups (*F*_6,14_ = 2.758, *p* = 0.055) (Fig. 8D). Nevertheless, the results of Spearman analysis showed a significant, moderate negative correlation between G721-0282 levels and VCAM mRNA (*R*=-0.51, *p* = 0.03) in spheroids, and this correlation was not observed in U-87 MG cells (Fig. 9). These results may indicate that inhibition of CHI3L1 has an effect in the context of the tumor tissue microenvironment, which may be missing when only 2D cell culture is analysed.

## Discussion

In this study, we analyzed the docking of G721-0282, the CHI3L1 protein inhibitor, to CHI3L1 protein and also performed molecular dynamics simulation for this molecule interactions. The results show that the complex of CHI3L1 and G721-0282 remains compact and stable. These results are a deeper insight into our previous docking studies on CHI3L1 and its inhibitor [15] as well as the studies by Park et al. [17] and the available data on CHI3L1-inhibitor interactions.

Molecular docking of CHI3L1 and G721-0282 was recently presented by Ham and coworkers [49]. In this work, G721-0282 was found to bind strongly to CHI3L1 among 14 million compounds with a binding affinity of − 7.2 kcal/mol [49]. Our results are consistent with these studies and show a similar binding affinity (–7.50 kcal/mol or − 7.81 kcal/mol, depending on the mode). The amino acids involved in the interaction of CHI3L1 and G721-0282 are also comparable in the studies of our team and Ham et al. However, in our work, we present for the first time a detailed interaction between the amino acids of CHI3L1 and the structure of G721-0282. In this article, we present the distribution of the different interaction energies for the CHI3L1-G721-0282 complex, which was previously unknown. In our in silico studies, we also used a molecular dynamics simulation (to our knowledge, no such calculations have been performed before). The stability of the system was demonstrated. A negative value of the binding free energy indicates a spontaneous process of complex formation. The van der Waals interaction energies for individual residues were also calculated.

Physical interactions between CHI3L1 and the inhibitor have so far only been demonstrated in a paper by Ham et al. in a pull-down assay and confirm the binding of these molecules [49]. Our results confirmed the binding between CHI3L1 and compound G721-0282 in ATR-FTIR spectroscopic analysis. Currently, there is no other work showing these direct interactions. In addition, theoretical calculations on the permeation of G721-0282 through BBB and low cytotoxicity in different cell lines were presented in our previous work [15]. Taken together, these results suggest this compound as a drug candidate for further investigation.

In a previous study by our team, we demonstrated the multidisciplinary effect of CHI3L1 achieved by G721-0282 [15]. These results show that the biological effect was mainly focused on the inhibition of angiogenesis in the in vitro HMEC-1 endothelial cell model and vascular mimicry in U-87 MG glioblastoma cells. In the spheroid model consisting of U-87 MG cells, HMEC-1 endothelial cells and macrophages, we also show an immune response of IL-1β, IL-6, IL-10, IL-18, TNF-α and β to the inhibitor of CHI3L1. In addition, we detected a decrease in the motility of U-87 MG glioblastoma due to a change in cytoskeletal architecture observed in digital holotomography (DHT). In short, changes indicative of inhibition of CHI3L1 by G721-0282 targeting the pSTAT-3 pathway.

The decreasing motility of U-87 MG cells observed in our previous studies needs further investigation in the context of the epithelial-mesenchymal transition (EMT) process, in which the balance between E-cadherin and N-cadherin plays an important role in GBM progression and response to treatment. Recently, research has shown that increasing expression of N-cadherin is associated with the resistance of GBM to radiotherapy. Mainly, this effect in glioblastoma stem cells (GCS) is responsible for the radioresistant phenotype of GCS induced by γ-irradiation in surviving cells, and this result has been shown in an in vivo model [50].

Higher expression of N-cadherin is associated with the anti-apoptotic effect of glioblastoma cells, and suppression of Wnt/β-catenin signaling leads to attenuated proliferation and neuronal differentiation [50]. The radioresistant phenotype of glioblastoma cells can be reversed by picropodophyllin, a clinically useful IGF-1 receptor inhibitor. Interestingly, Francescone’s team [49] observed an increased expression of CHI3L1 in U-87 MG glioblastoma cells after γ-irradiation, leading to protection against cell death. In experiments with γ-irradiation, the authors also observed a regulatory role of CHI3L1 for VEGF [51] and we also observed this relationship in our preliminary study with a CHI3L1 inhibitor performed on a spheroid model of GBM [15].

The role of CHI3L1 in the extracellular matrix (ECM) is currently known to be related to the inhibition of metalloproteinases (MMPs) by this protein and influences the motility and invasiveness of cancer cells [13]. CHI3L1 decreased the expression of E-cadherin, which leads to a reduction in cell adhesion and may promote cell migration in breast cancer cells. The effect of CHI3L1 on reducing E-cadherin expression and simultaneously increasing MMP-9 was demonstrated in a study of breast cancer cells by Scully and colleagues [52]. In our studies, we did not observe changes in E-cadherin levels in either U-87 MG glioblastoma cells or GBM spheroids. In the experiments performed by Osuka et al., γ-irradiation led to a decrease in N-cadherin levels, but E-cadherin levels remained unchanged [50]. In fact, the expression of E-cadherin is rather rare in GBM [53]. Nevertheless, recent therapies point to the possible role of oncolytic viruses leading to overexpression of this cadherin in GBM cells [54]. Our results confirm the separate mechanism of regulation of this cadherin achieved by inhibition of CHI3L1 in our studies, suggesting that highest concentration of G721-0282 leads to a reduction of N-cadherin expression in our spheroid model of GBM at the protein level in immunoblotting and immunohistochemical reaction as well as at the mRNA level. Interestingly, increased expression of E-cadherin and N-cadherin was observed in spheroids treated with 50 mM of G721-0282 at the protein level, but not at the mRNA level. This effect may suggest that the window of action for CHI3L1 inhibition by G721-0282 is narrow and, secondly, that some compensatory effect may occur at lower concentration of inhibitor. Also, we postulate, that CHI3L1 inhibition with G721-0282 may not occurs linearity, we also observed this effect in our previous studies conducted by our team [15]. It is known that inhibition of protein functionality can lead to increased expression of the target protein, as we observed in this study in the case of CHI3L1 and also in our previous study of CHI3L1 inhibition with G721-0282 [15]. The biological effect of the change in cadherin expression as a result of CHI3L1 inhibition is novel and worthy of further investigation. Currently, N-cadherin is one of the potential targets, as lower levels of this protein prolong patient survival [55]. In our study, we found a difference between the results of the in vitro model and the spheroids. In contrast to the results obtained in the spheroids, an increase in N-cadherin levels was observed in the traditional two-dimensional culture of U-87 MG glioblastoma cells. This result suggests that the spheroid model is more suitable for the study of cell-cell interactions. The balance between cadherins plays a crucial role in carcinogenesis, especially in the mechanisms that regulate cancer progression. Higher expression of N-cadherin compared to E-cadherin is termed epithelial-mesenchymal transition (EMT) and results in loss of adhesion between cells, leading to increased motility, invasiveness, and metastasis [56]. EMT factors are commonly involved in this process and are mostly labeled as ZEB1, Twist, Snail, and Slug [57].

EGFR (epidermal growth factor receptor) expression, in turn, is upregulated in about 60% of glioblastoma cases and is associated with tumor progression and γ-radiation resistance [57]. The EGFRIII isoform causes permanent activation of this receptor and transmits the activation signal to common signaling pathways involved in carcinogenesis, including JAK/STAT, PI3K/Akt, and ERK1/2 [25, 58, 59]. Inhibition of EGFR has clinical utility, and EGFR inhibitors such as erlotinib and gefitinib are novel therapeutic strategies [8].

In addition, studies by Zheng and coworkers show that EGFR activation is associated with an increase in VCAM-1, leading to increased macrophage infiltration [59]. Recent studies have also shown that cytokines, particularly IL-1β, increase the expression of the adhesion molecules ICAM-1 and VCAM-1 in GBM cells [60]. Our research has shown decreasing in VCAM-1 expression in GBM spheroids and also in U-87 MG glioblastoma cells to G721-0282 treatment, mainly at concentrations of 100 − 25 µM, which could be explained by VCAM-1/IL-1β dependence. In previous studies, a decrease in IL-1β expression levels upon treatment with G721-0282 was observed in this same type of GBM spheroids [15]. This suggests a broad spectrum of anti-cancer activity associated with CHI3L1 inhibition. In the literature, the effect of a higher concentration of IL-1β is also associated with increased adhesion of monocytes to GBM tumor cells [60]. This effect could potentially be useful in multicenter therapy involving cell adhesion and immunomodulatory background of GBM tumor components. Recently, Cheng et al. [61] proposed the use of VCAM-1 in MRI techniques to delineate the tumor-brain interface.

In this study, the new label-free physical cytometer techniques were used to evaluate the uptake of inhibitors into spheroids. The results showed that the highest concentration of G721-0282 penetrated best into the spheroids, which explains the observed biological effect at 100 µM of the inhibitor. Moreover, these techniques allowed the observation of the absence of cytotoxic effects of the inhibitor, which was shown in our previous research on GBM spheroids and also separately on each cell line that was part of it, indicating a specific and selective effect of this inhibition [15]. To our knowledge, this is the first study to present the use of a physical cytometer to analyze CHI3L1 inhibition. It is also the first study to present the biological effect of this inhibition in GBM spheroids in the context of changes in adhesion protein expression.

Our research is a continuation of the exploration of the potential of CHI3L1 inhibition in the development of new targeted therapies for GBM for future clinical applications. The spheroid model used in this study can be successfully used to answer the most fundamental question and serve as a bridge between in vitro and in vivo experiments.

## Conclusions

Our research has shown that the CHI3L1 protein interacts with its inhibitor, the compound G721-0282, and forms a stability complex. In addition, G721-0282 is able to penetrate into GBM spheroids and affect the expression of adhesion proteins. At the biological level, inhibition of CHI3L1 leads to a decrease in VCAM-1 and N-cadherin. This results suggesting a potential novel approach for targeted therapy of GBM.

## Electronic supplementary material

Below is the link to the electronic supplementary material.

Supplementary Material 1

Supplementary Material 2

## Data Availability

The raw data supporting the conclusions of this article will be made available by the authors on request.

## Abbreviations

GBM: Glioblastoma multiforme
BBB: Blood-brain barrier
TMZ: Temozolomide
MS: Multiple sclerosis
VEGF: Venous epithelial growth factor
CHI3L1: Chitinase-3- like protein 1
EGFR: Epidermal growth factor receptor
VCAM-1: Vascular cell adhesion molecule 1
DMSO: Dimethyl sulfoxide
PMA: Phorbol 12-myristate 13-acetate
FGF: Fibroblast growth factor
(ATR-FTIR: Fourier transform infrared spectroscopy
ESEM: Environmental scanning electron microscope
RIPA: Radioimmunoprecipitation assay buffer
BSA: Bovine serum albumin
EDTA: Ethylenediaminetetraacetic acid
PMSF: Phenylmethanesulfonyl fluoride
BCA: Bicinchoninic acid
DHT: Digital holotomography
EMT: Epithelial-mesenchymal transition
JAK: Janus kinase
STAT: Signal transducer and activator of transcription proteins
PI3K: Phosphatidylinositol-3-kinase
Akt: Protein kinase B
ERK1/2: Extracellular signal-regulated kinase 1/2
