# Supplementary material for: Inhibition of CHI3L1 decreases N-cadherin and VCAM-1 levels in glioblastoma
Source: Pharmacol Rep. 2024 Nov 14;77(1):210–28. doi: 10.1007/s43440-024-00677-3 (PMC11743419; doi:10.1007/s43440-024-00677-3)
Supplement: Supplementary file 1 — Supplementary Material 1 [file 43440_2024_677_MOESM1_ESM.pdf]

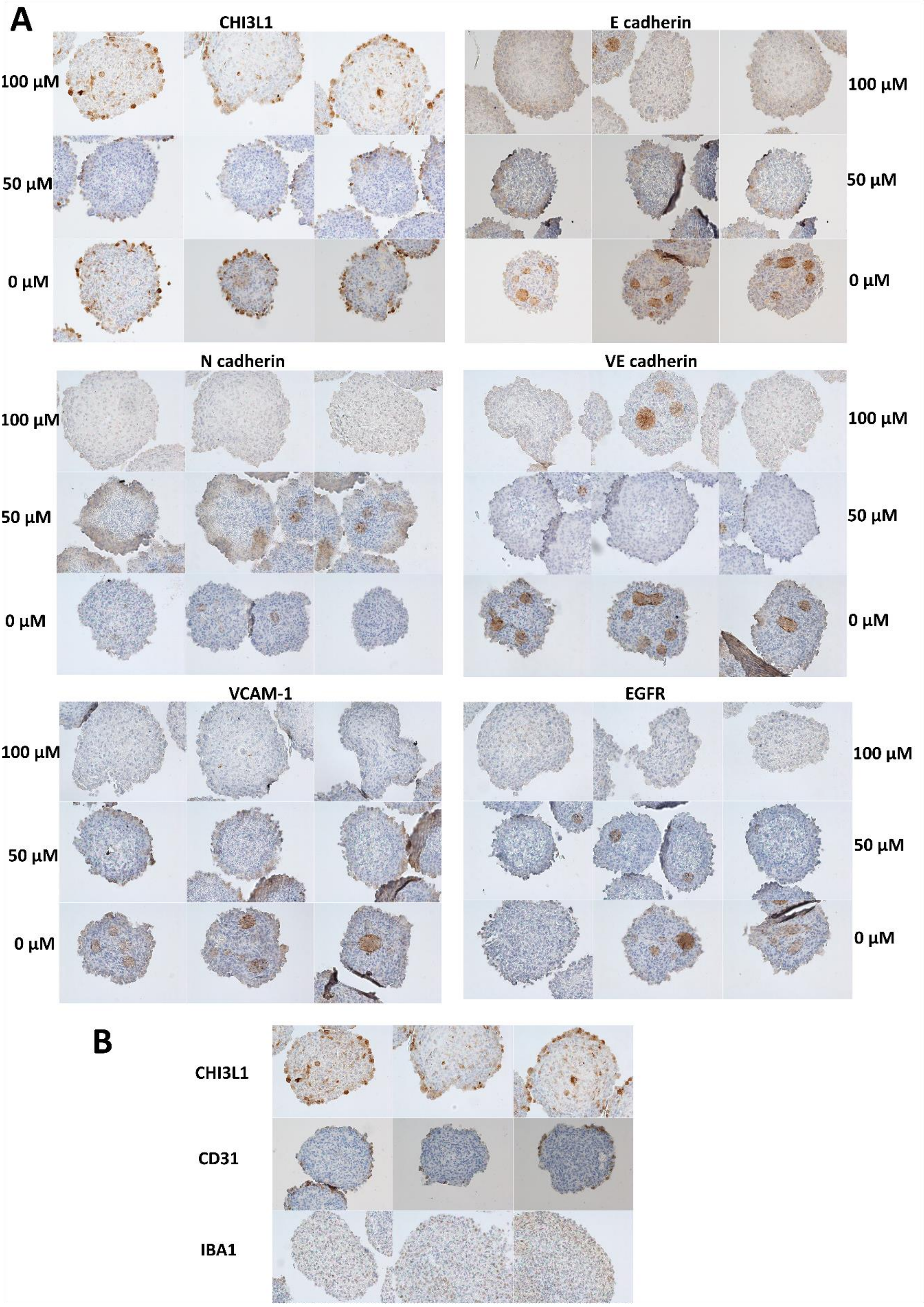

Supplementary Figure S1. Examples of immunohistochemical images of spheroids. Panels of images (A) showed expression level and localization of CHI3L1, cadherins, VCAM-1 and EGFR after treatment with G721-0282 compound (the inhibitor of CHI3L1) in concentration 100 and 50 µM and also in culture medium without inhibitor (0 µM); (B) typical markers expression were analyzed using immunohistochemical reactions in U-87 MG glioblastoma cells (CHI3L1-possitive), endothelial cells HMEC-1 (CD31-positive) and macrophages (IBA1-positive), to confirm three cell components of spheroids used in the study; light microscope BX41 (Olympus); magn.100x*;* CHI3L1: chitinase-3- like protein 1; EGFR: epidermal growth factor receptor; VCAM-1: vascular cell adhesion molecule 1.
