# Supplementary material for: Inhibition of CHI3L1 decreases N-cadherin and VCAM-1 levels in glioblastoma
Source: Pharmacol Rep. 2024 Nov 14;77(1):210–28. doi: 10.1007/s43440-024-00677-3 (PMC11743419; doi:10.1007/s43440-024-00677-3)

Supplementary Figure S2. Raw images of Western blots membranes. Analysed material: U-87 MG glioblastoma cells and spheroids consisted od U-87 MG, HMEC-1 endothelial cells and macrophages. G721-0282 compound (the CHI3L1 inhibitor) was used in concentration range (100-6.25 µM). Legend: 1-5 diffrent concentrations of G721-0282; 1:100µM, 2: 50 µM, 3: 25 µM, 4:12.5 µM, 5:6.25 µM; 6: Solvent control (spheroids cultured in medium with addition of 0.1% DMSO (the solvent for G721-0282) occurred at concentration of 100 mM of G721-0282), 7: control (culture medium only) visualization was provide with the ChemiDoc TM MP system (Bio-Rad) and ImageLab software (Bio-Rad).

CHI3L1: 40 kDa

U-87 MG cells

GBM spheroids

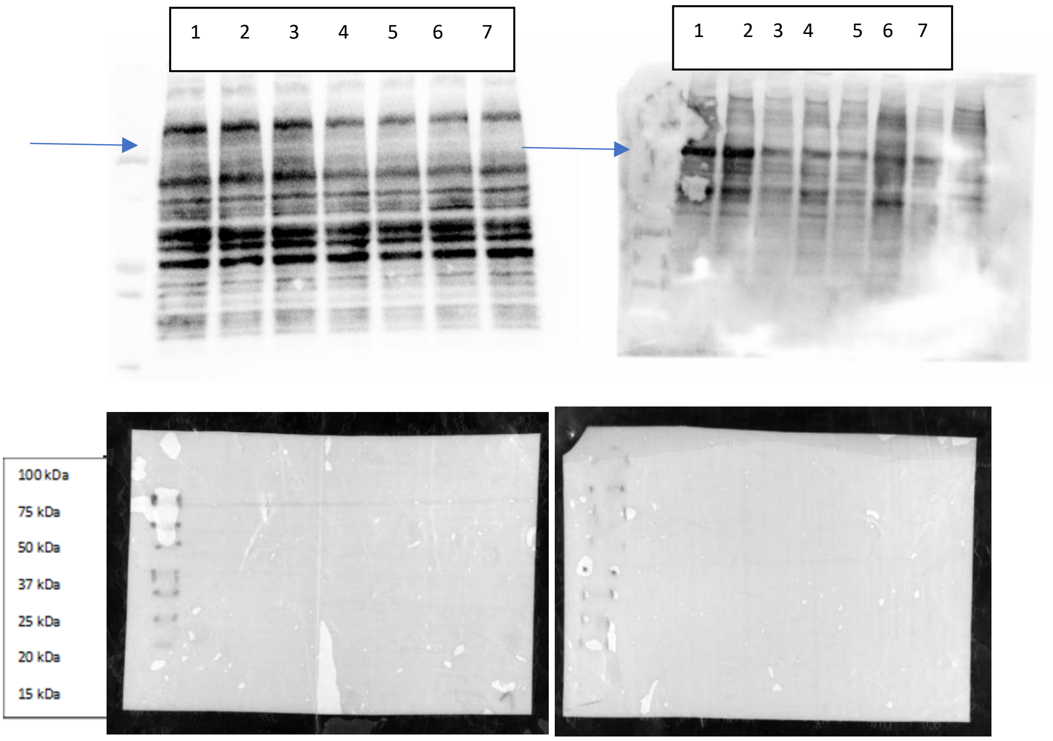

N-cadherin: 134 kDa

U-87 MG cells

GBM spheroids

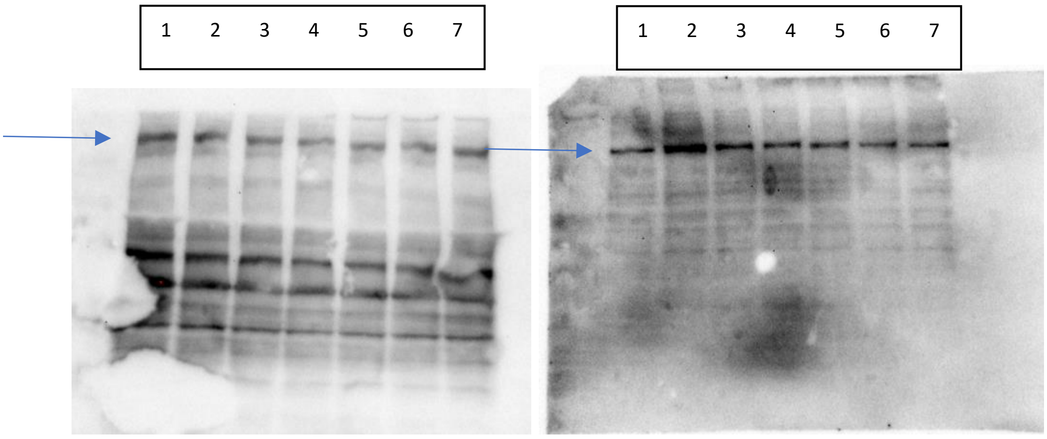

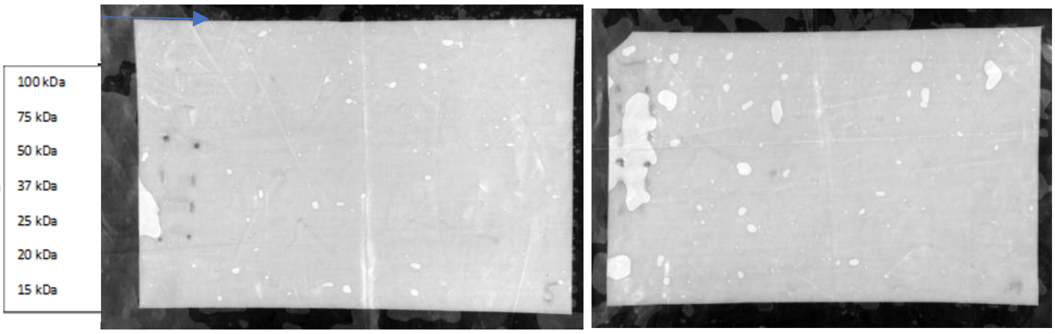

E-cadherin: 130 kDa

U-87 MG cells

GBM spheroids

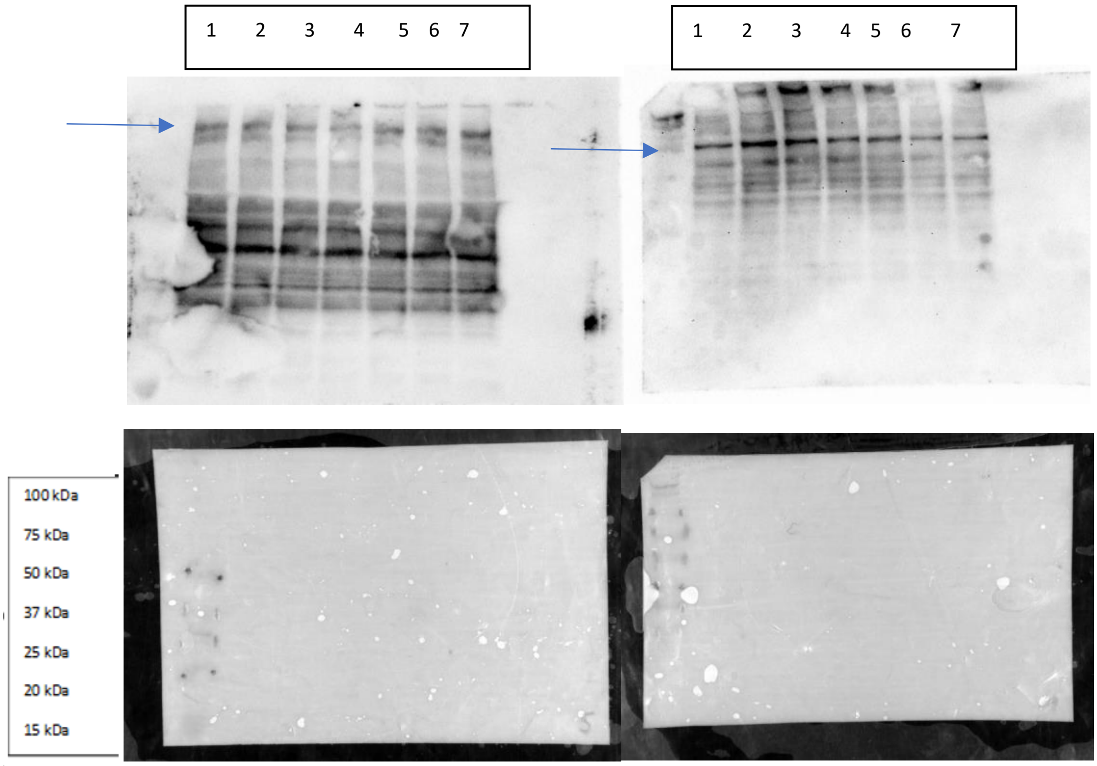

VCAM-1: 80 kDa

U-87 MG cells   GBM spheroids

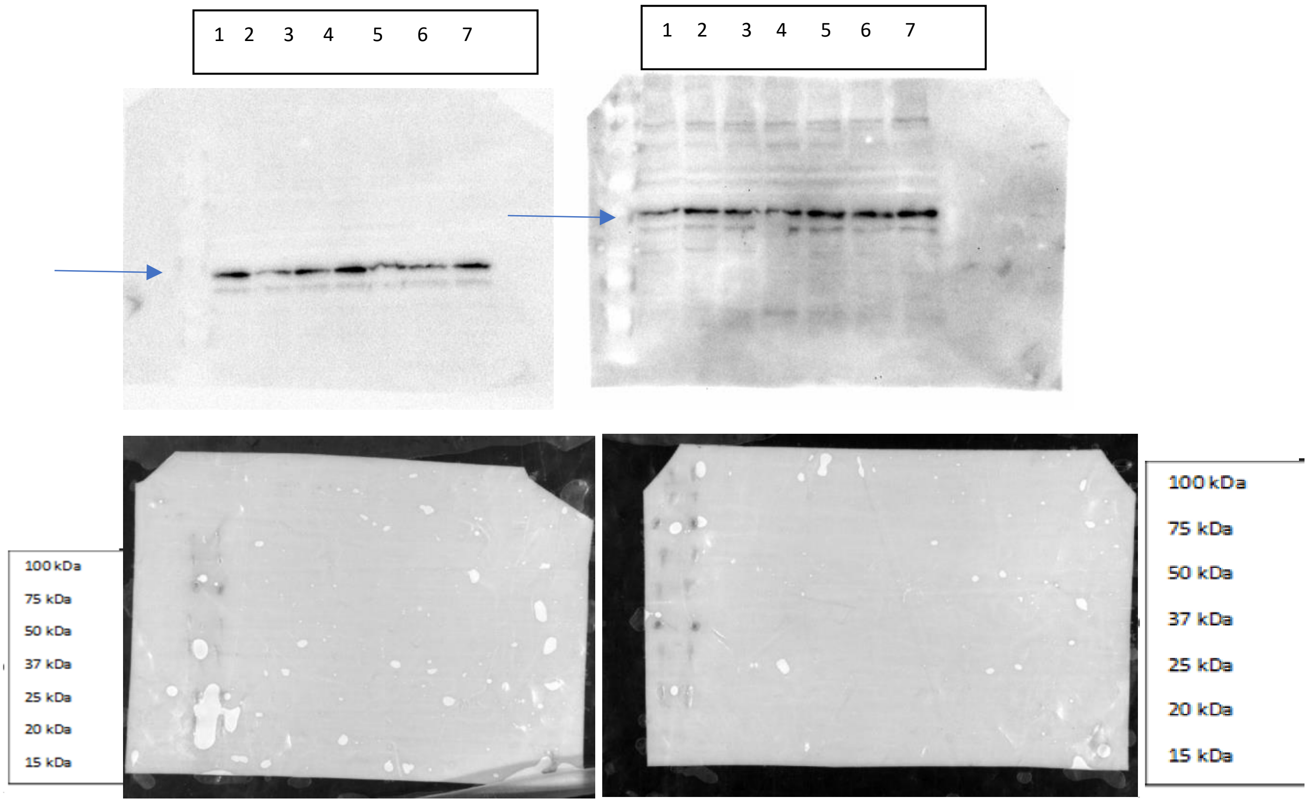

VE-cadherin : 132 kDa

U-87 MG cells   GBM spheroids

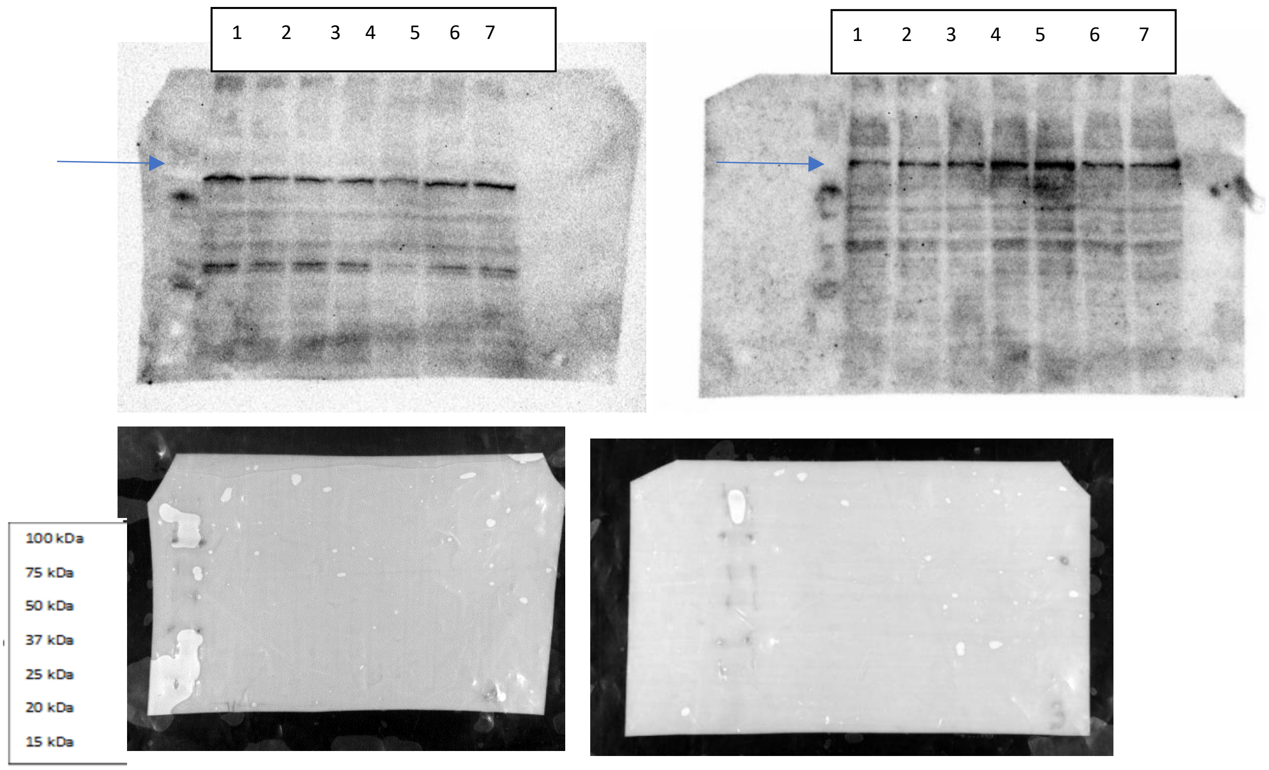

GAPDH: 36 kDa

U-87 MG cells

GBM spheroids

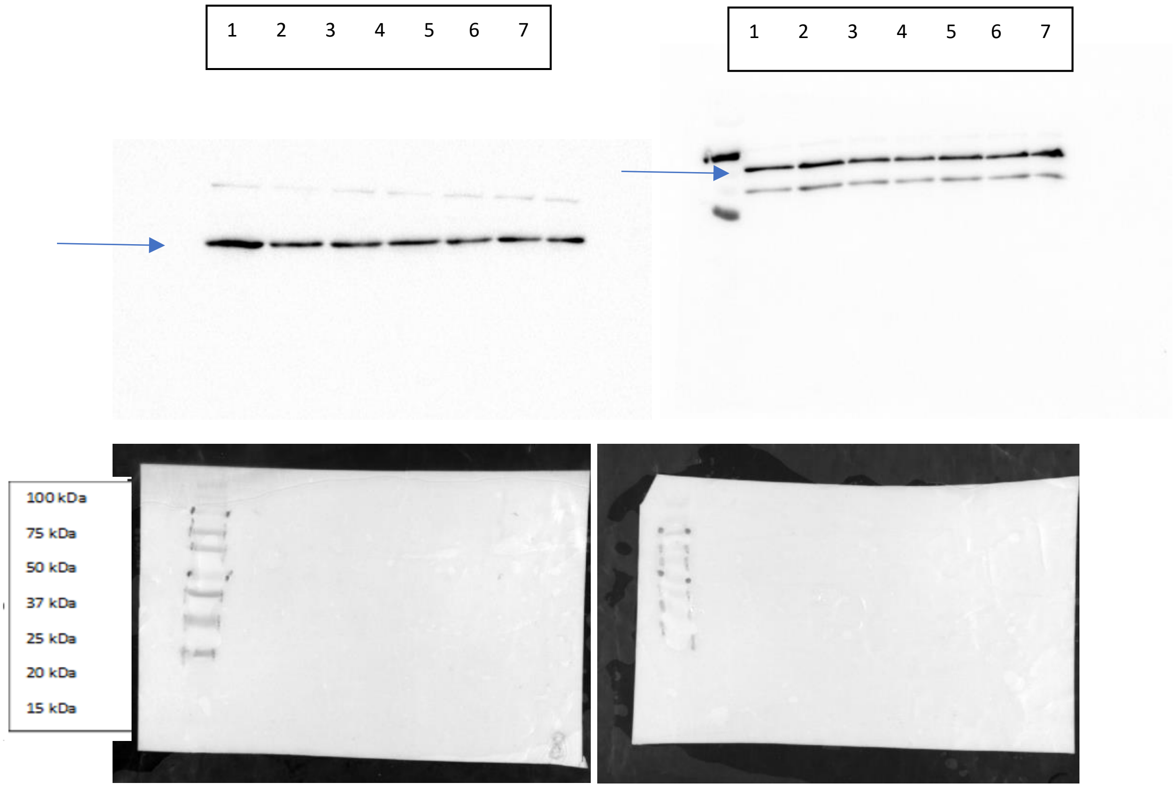

β-Tubulin: 50 kDa

U-87 MG cells

GBM spheroids

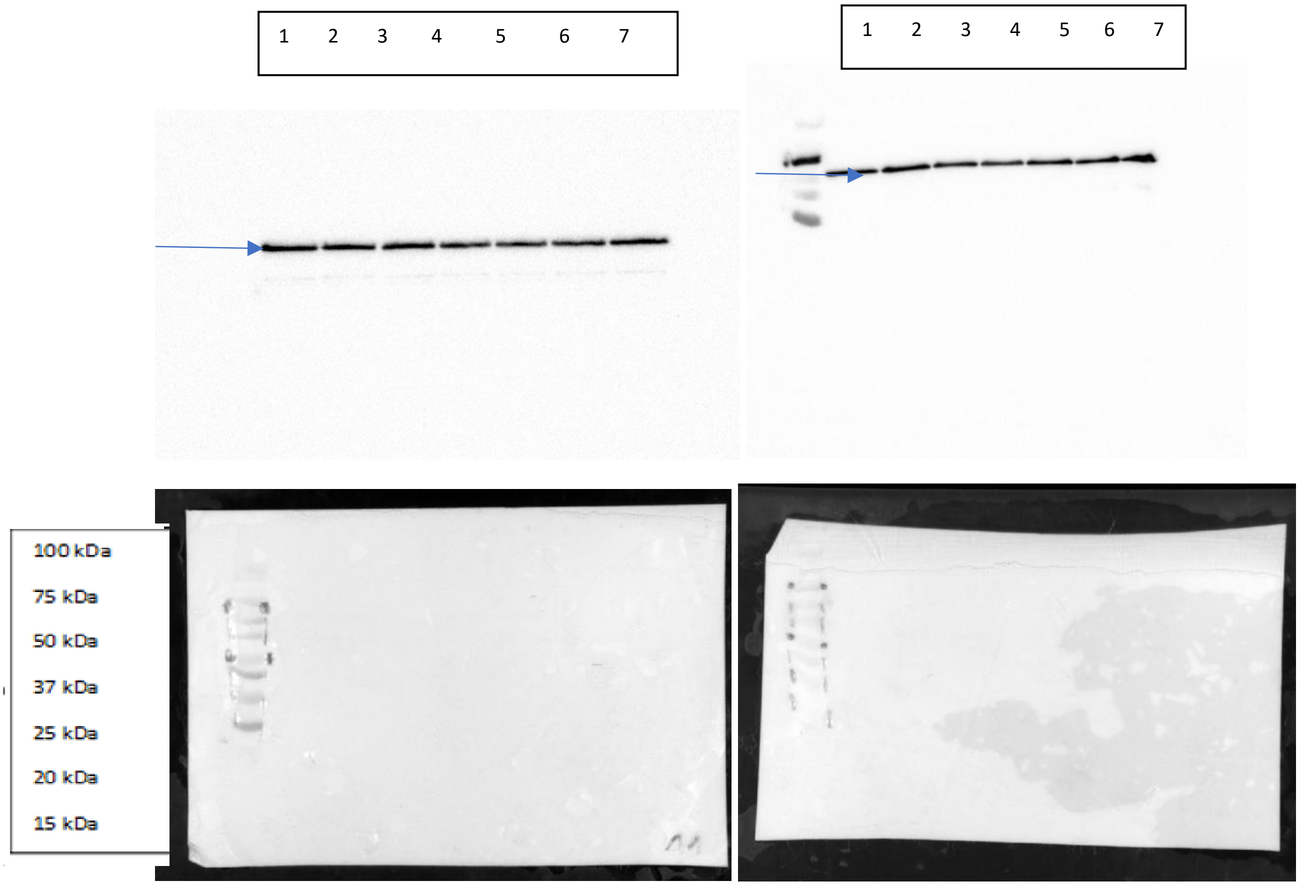

Supplement: Supplementary file 2 — Supplementary Material 2 [file 43440_2024_677_MOESM2_ESM.pdf]
